# Microbial Interactions and Flavor Modulation in Cabbage Fermentation: Roles of *Lactiplantibacillus plantarum* and *Rhodotorula mucilaginosa*

**DOI:** 10.3390/molecules31152666

**Published:** 2026-07-30

**Authors:** Jiaqian Liang, Shiying Zeng, Tao Wang, Chuanqi Chu, Junjie Yi, Zhijia Liu

**Affiliations:** 1 Faculty of Food Science and Engineering, Kunming University of Science and Technology, Kunming 650500, China; 2 Yunnan Key Laboratory of Plateau Food Intelligent Processing, Kunming 650500, China; 3 International Green Food Processing Research and Development Center of Kunming City, Kunming 650500, China

**Keywords:** *Lactiplantibacillus plantarum*, *Rhodotorula mucilaginosa*, vegetable fermentation, flavor, microbial correlation analysis

## Abstract

Fermented cabbage is a globally popular food, whose distinctive flavor is largely determined by microbial metabolism and interspecies interactions. To understand the roles of *Lactiplantibacillus plantarum* (formerly *Lactobacillus plantarum*) and *Rhodotorula mucilaginosa* in regulating microbial community structure and flavor formation during cabbage fermentation, natural fermented cabbage was compared with that inoculated with *L. plantarum*, *R. mucilaginosa* and both strains. The results showed that cabbage inoculated with either strain accelerated fermentation, with the fastest acidification observed in the co-inoculated group. Additionally, cabbage inoculated with *R. mucilaginosa* exhibited a relatively higher total amino acid content (481.50 ± 7.75 g/kg), and the levels of sweet, bitter, and umami amino acids were significantly higher than in the other groups. At the final fermentation stage, 15 differential odor-active compounds (ROAV ≥ 0.1, VIP ≥ 0.5) were identified, contributing mushroom, floral, fruity, malty, buttery, fatty, citrus, soapy, and green notes. Microbial succession analysis revealed that *L. plantarum* dominated bacterial community restructuring during fermentation and showed antagonistic relationships with the other lactic acid bacteria, including *Lactococcus* and *Weissella*. In contrast, *R. mucilaginosa* mainly influenced fungal community succession and indirectly modulated flavor formation by regulating the abundance of flavor-associated microorganisms, including *Lactiplantibacillus*, *Lactococcus*, *Staphylococcus*, *Debaryomyces*, and *Candida*. Correlation analysis further suggested that *L. plantarum* functioned as a major flavor-driving microorganism; whereas, *R. mucilaginosa* acted primarily as a microbial community modulator that enhanced flavor complexity through interspecies interactions. These findings highlight the importance of microbial interactions in shaping the flavor characteristics of fermented cabbage and provide insights for improving fermented vegetable flavor through targeted microbial regulation.

## 1. Introduction

Fermented vegetables, a major category of fermented foods, originated in ancient China as a traditional strategy to enhance the shelf stability of fresh vegetables [1,2]. Fermented vegetables exhibit distinct regional characteristics, partly reflected by the use of different base ingredients across regions, among which cabbage is one of the most widely used raw materials in vegetables ferments [2,3]. Within vegetable fermentation, interactions between microorganisms and substrates are indispensable for the metabolic activities and formation of distinctive flavors [4].

Vegetable matrices are degraded by microbial metabolism into various small-molecule compounds, including free amino acids, alcohols, aldehydes, and organic acids [5]. These primary metabolites subsequently participate in esterification reactions within the fermentative system, leading to the formation of esters [6]. Various genera of lactic acid bacteria (LAB) have been identified as the dominant members in most fermented vegetables [7]. Beyond their role in acidification, which facilitates fermentation progression and suppresses undesirable microorganisms, LAB are also important contributors to flavor formation [8,9]. Specifically, LAB participate in the production of amino acids, peptides, and organic acids, thereby shaping the flavor profile of fermented vegetables [6]. In addition to LAB, yeasts, although typically present as non-dominant microorganisms in fermented vegetables, pivotally contribute to flavor formation by correlating with the accumulation of taste-active free amino acids, alcohols, and esters that impart pleasant taste attributes and fruity or floral aromas [9]. Hence, the metabolic activities of LAB and yeasts define the overall flavors of fermented vegetables.

Starters are widely used to achieve controlled and reliable fermentation processes [4]. However, single-strain starters may fail to construct sufficient flavor complexity of fermented food, prompting the application of mixed cultures, as co-fermentation may enrich both non-volatile and volatile compounds [10,11]. Apart from flavor intensification, yeasts interact with LAB and may modulate their growth dynamics [12]. For instance, yeasts may support LAB growth by releasing nutrients through the degradation of complex substrates, or by alleviating growth stress via the removal of inhibitory compounds [2]. Moreover, certain microbial strains may alter the behavior of co-occurring microbes, thereby generating unique flavors and esthetics [13]. Although mixed starter cultures have been specified to enhance the flavor of fermented vegetables, the synergy of mixed starters on the entire fermentative ecosystem and their action mode underlying flavor development remain poorly understood. Therefore, it is necessary to investigate the roles of inoculated starters in flavor formation and massive microbe dynamics within vegetable ferments.

*L. plantarum* and *R. mucilaginosa* were selected as the representatives for LAB and yeast, respectively, since *L. plantarum* is a core species in fermented vegetables [14] and *R. mucilaginosa* exhibits capacity for flavor optimization [15]. In our previous study, co-fermentation with *L. plantarum* and *R. mucilaginosa* in a sterile-based simulated system initially elucidated their interactions in terms of proliferation regulation and flavor enhancement [12]. In the present study, these two strains were inoculated to conduct the actual cabbage fermentation. The outcomes of inoculated fermentation were compared with those of spontaneous fermentation by monitoring the dynamics of physicochemical parameters and flavor substances, including pH, acidity, organic acids, free amino acids and volatile compounds, throughout fermentation. Furthermore, combined with high-throughput sequencing and correlation analysis, microbial profiles were integrated to investigate both strains’ joint effects on flavor development and microbial constitution which in return exerts influence on the flavor profiles. The objective of this study was to deeply understand the synergistic effects of LAB and yeast within the cabbage fermentation ecosystem and their roles in flavor modulation.

## 2. Results

### 2.1. Changes in Total Sugars, pH and Acidity

**Figure 1:**
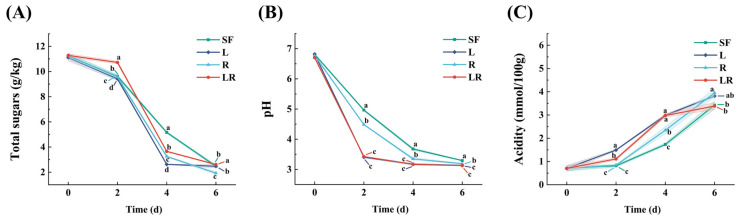
Changes in physicochemical indicators during FC: (**A**) total sugars, (**B**) pH, (**C**) acidity. Different lowercase letters indicate significant differences among SF (spontaneous fermentation), L (*L. plantarum* inoculation), R (*R. mucilaginosa* inoculation), and LR (Co-inoculation of *L. plantarum* and *R. mucilaginosa* at a 1:1 ratio) at the same fermentation stage according to Tukey test (*p* < 0.05). Data are presented as mean ± standard deviation (SD). The differences indicated by different letters were statistically significant (*p* < 0.05).

During cabbage fermentation, polysaccharides such as cellulose are hydrolyzed by microorganisms into low-molecular-weight sugars, which are subsequently utilized to sustain microbial growth and metabolism [16]. As shown in Figure 1A, fermentation approached completion by 6 d, as total sugars remained relatively stable between days 4 and 6. In all inoculated groups, *L. plantarum* inoculation (L), *R. mucilaginosa* inoculation (R), and co-inoculation of *L. plantarum* and *R. mucilaginosa* at a 1:1 ratio (LR), total sugars were rapidly depleted during the mid-fermentation stage (2–4 d), reaching significantly lower levels than in spontaneous fermentation (SF) by 4 d. Notably, the R group exhibited the lowest total sugar content at the end of fermentation, implying that inoculation with *R. mucilaginosa* enhanced sugar utilization. Acidification reflects the progression of fermentation (Figure 1B,C). The pH in all groups decreased rapidly from day 0 to 4 d; however, the rates of decline differed among groups, following the order LR = L > R > SF. In parallel, acidity accumulation followed the trend L > LR > R > SF. Acidity increased slowly during the early stage (0–2 d) but rose sharply from 2 d to 6 d, reaching final values of 3.95 ± 0.05 mmol/100 g (R), 3.82 ± 0.05 mmol/100 g (L), 3.42 ± 0.17 mmol/100 g (SF), and 3.39 ± 0.16 mmol/100 g (LR). Collectively, these findings demonstrate that inoculation with *L. plantarum* accelerated the fermentation process of FC; whereas, the R group (inoculated with *R. mucilaginosa*) exhibited greater total acidity accumulation, mainly composed of organic acids, than the co-inoculated LR group.

### 2.2. Dynamics of Organic Acids

**Figure 2:**
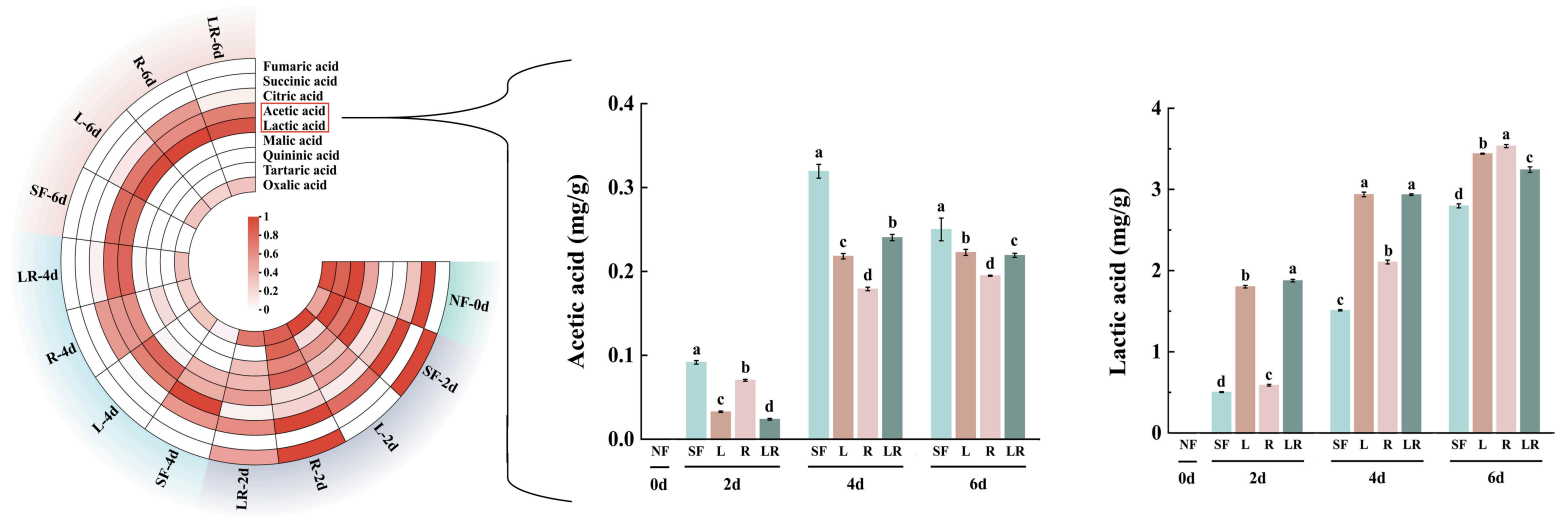
Heatmap of organic acid profiles and bar plots of acetic acid and lactic acid during FC fermentation. SF (spontaneous fermentation), L (*L. plantarum* inoculation), R (*R. mucilaginosa* inoculation), and LR (Co-inoculation of *L. plantarum* and *R. mucilaginosa* at a 1:1 ratio). The differences indicated by different letters were statistically significant (*p* < 0.05).

Microorganisms regulate organic acid metabolisms to sustain cellular functions, thereby shaping the acidity and flavor profile of fermented foods [17,18]. During cabbage fermentation, most organic acids showed a declining trend, including fumaric, citric, malic, quinic, tartaric, and oxalic acids; whereas, lactic acid and acetic acid exhibited the opposite pattern (Figure 2). The reduction in oxalic acids is associated with improved taste quality of FC, as it is known to impart astringent notes. Quinic acid serves as an important precursor in flavor formation [19]. It participates in the shikimate pathway leading to phenylalanine synthesis, which can be further converted into phenethyl alcohol, contributing floral and fruity aromas [20]. Notably, quinic acid levels in the inoculated groups were lower than those in SF during 0 to 2 d, implying that inoculation promoted its utilization, thereby supporting aroma precursor formation. Lactic acid was the predominant organic acid, contributing substantially to taste and reflecting fermentation progression; whereas, acetic acid provides sourness and pungency that are characteristic of fermented vegetables [21,22]. During fermentation, acetic acid in SF increased sharply to 0.31 ± 0 mg/g on 4 d and then decreased to 0.25 ± 0.01 mg/g on 6 d. In contrast, the inoculated groups accumulated acetic acid at day 4 but maintained relatively stable levels thereafter (Figure 2 and Table S1). This pattern demonstrates that inoculation altered acetic acid accumulation, particularly at the late stage of fermentation.

Among all groups, R showed the highest lactic acid content at 3.53 ± 0.01 mg/g, followed by L, LR, and SF (Figure 2 and Table S1). Previous studies have shown that yeasts possess enzymatic systems capable of degrading macromolecules into smaller compounds, thereby supplying substrates for LAB growth and metabolism [23]. *R. mucilaginosa* has also been reported to promote the proliferation of *L. plantarum* in co-culture systems, leading to enhanced lactic acid production [12]. In this study, the higher lactic acid level in R compared with LR implies that *R. mucilaginosa* may also stimulate indigenous LAB, particularly strains with strong lactic acid producing capacity, which could explain the elevated lactic acid accumulation in the R group.

### 2.3. Free Amino Acid (FAA) Dynamics

**Figure 3:**
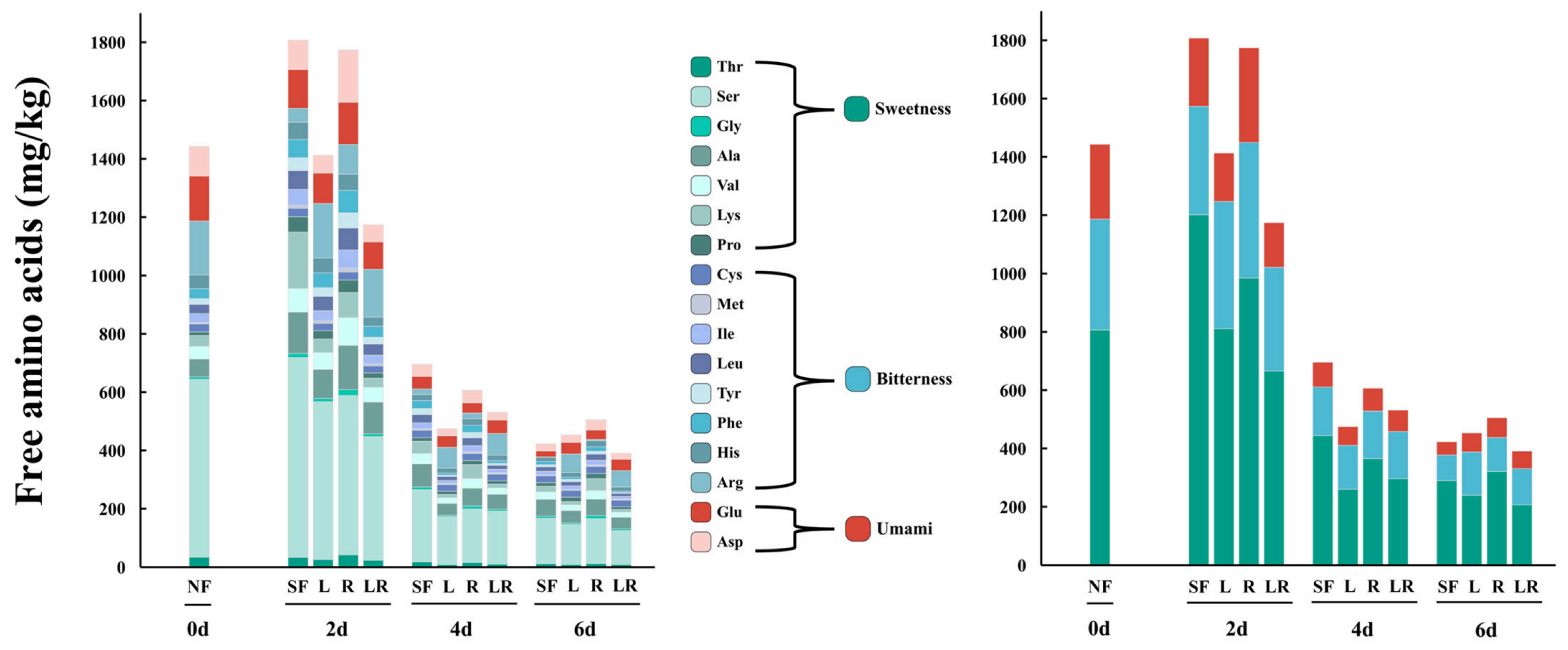
Changes in free amino acids concentration in different FC groups. SF (spontaneous fermentation), L (*L. plantarum* inoculation), R (*R. mucilaginosa* inoculation), and LR (Co-inoculation of *L. plantarum* and *R. mucilaginosa* at a 1:1 ratio). Thr (Threonine); Ser (Serine); Gly (Glycine); Ala (Alanine); Val (Valine); Lys (Lysine); Pro (Proline); Cys (Cysteine); Met (Methionine); Leu (Leucine); Ile (Isoleucine); Tyr (Tyrosine); Phe (Phenylalanine); His (Histidine); Arg (Arginine); Glu (Glutamic acid); Asp (Aspartic acid).

Free amino acids are essential nutrients for microbial growth and metabolism and are key contributors to the taste profile of fermented foods [24]. As shown in Figure 3, total FAA concentrations increased from day 0 to 2 d and then declined progressively from 2 d to 6 d, reflecting active microbial metabolism and subsequent utilization of amino acids. At the end of fermentation, the R exhibited the highest levels of sweet FAAs (321.00 ± 3.00 mg/kg), umami FAAs (67.50 ± 0.50 mg/kg), and total FAAs (505.50 ± 1.50 mg/kg) compared with the other groups (Figure 3 and Table S2). This result demonstrates that inoculation with *R. mucilaginosa* promoted the formation of taste-active amino acids. Yeasts are known to possess efficient enzymatic systems that hydrolyze macromolecules into smaller amino acids, thereby enriching the FAA pool [23]. Notably, phenylalanine in the R reached the highest level on 2 d at 77.00 ± 0 mg/kg and decreased to 15.50 ± 0 mg/kg by 6 d, reflecting its conversion into aromatic compounds such as phenethyl alcohol. Umami FAAs mainly consist of aspartic acid and glutamic acid, which enhance the savory taste of FC [25]. The R showed the highest concentration of aspartic acid at 35.50 ± 0.40 mg/kg; whereas, the L exhibited the highest glutamic acid content at 39.50 ± 0.40 mg/kg at the end of fermentation. These observations demonstrate that single-strain inoculation with *R. mucilaginosa* or *L. plantarum* promoted the accumulation of umami amino acids, thereby enhancing flavor complexity and palatability. However, the co-inoculated group (LR) did not show superior accumulation of aroma-active FAAs compared with the other groups. This outcome can be attributed to the accelerated fermentation and intensified metabolic activity in LR due to synergistic effect by the two strains, which increased the consumption of FAAs as nitrogen sources for microbial growth and as precursors for volatile compound formation (Figure 3).

### 2.4. Analysis of Volatile Compounds in FC

**Figure 4:**
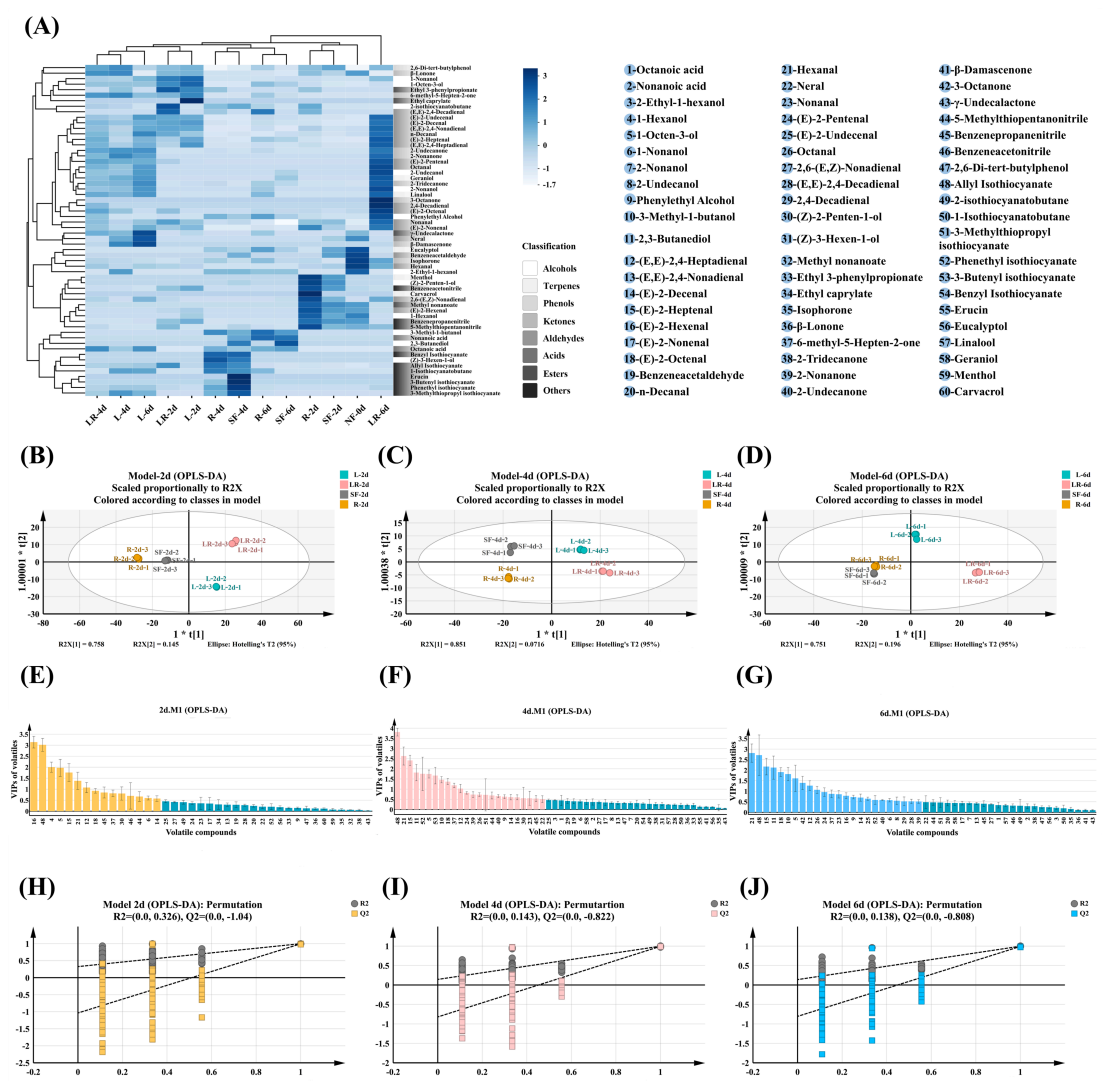
(**A**) Comprehensive profiling of volatile compounds and identification of differential odor-active compounds in FC. (**A**) Concentration distribution of volatiles visualized by heatmap; (**B**–**D**) screening of differential odor-active compounds (ROAV ≥ 0.1, VIP ≥ 0.5) in different FC samples using OPLS-DA, supported by (**E**–**G**) VIP scoring and (**H**–**J**) permutation testing. SF (spontaneous fermentation); L (*L. plantarum inoculation*), R (*R. mucilaginosa inoculation*), and LR (Co-inoculation of *L. plantarum* and *R. mucilaginosa* at a 1:1 ratio).The dash lines indicated the linear regression of the corresponding R^2^ and Q^2^ values obtained from the 200 permutation tests.

A total of 60 volatile compounds were identified in FC, including 11 alcohols, 5 terpenes, one phenol, eight ketones, 18 aldehydes, two acids, four esters, eight sulfides, and three other compounds (Table S3). The average clustering heatmap (Figure 4A) shows that the volatile profiles of SF and R at 2 d were similar to those at day 0, reflecting a relatively low degree of fermentation at the early stage. This pattern is consistent with the slower pH decline observed in these groups (Figure 1B). By 6 d, LR exhibited a distinct volatile profile compared with the other groups, as clustering separated LR from all others, demonstrating that co-inoculation with *L. plantarum* and *R. mucilaginosa* markedly altered the final aroma composition. To further resolve differences among samples and identify key discriminatory volatiles, OPLS-DA was conducted based on the concentrations of the 60 compounds (Figure 4B–D). The model evaluation parameters were R2X = 0.99, R2Y = 0.996, Q2 = 0.987 at 2 d; R2X = 0.98, R2Y = 0.992, Q2 = 0.983 at 4 d; and R2X = 0.985, R2Y = 0.989, Q2 = 0.983 at 6 d, confirming strong explanatory and predictive performance. VIP values were then used to identify the differential volatiles among different groups (Figure 4E–G). Permutation testing with 200 iterations and Q2 regression analysis confirmed model validity and excluded overfitting (Figure 4H–J).

The score plots clearly separated SF, L, R, and LR across fermentation stages (Figure 4B–D), although R and SF were positioned closely at 6 d, reflecting similarity in their volatile compositions. Based on VIP values, 15, 24, and 24 differential volatile compounds (VIP ≥ 0.5) were identified at 2 d, 4 d, and 6 d, respectively (Figure 4E–G). Notably, 18 differential compounds detected from 4 d to 6 d belonged mainly to alcohols, aldehydes, ketones, and sulfides, contributing sensory attributes such as mushroom-like, floral, malty, buttery, fatty, green, soapy, citrus-like, garlic-like, and boiled vegetable-like notes (Figure 4E–G and Table S4). These compounds likely drive the divergence in aroma profiles among groups during the middle and late stages of fermentation. ROAV analysis was applied to identify key odor-active compounds (Table S4). A total of 33 volatiles were classified as odor-active compounds with ROAV ≥ 0.1. When combined with VIP analysis, 15 differential odor-active compounds (VIP ≥ 0.5) were identified at the final stage, contributing notes such as mushroom-like, floral, fruity, malty, buttery, fatty, citrus-like, soapy, and green characteristics.

Among the compounds distinguishing groups at 6 d, (E)-2-decenal and nonanal contributed green and fatty notes commonly associated with lipid oxidation and undesirable aromas due to their low odor thresholds. Although their concentrations were higher in the inoculated groups than in SF, their ROAVs were lower, demonstrating that their sensory impact was reduced in the inoculated samples (Tables S3 and S4). 2-Nonanone, which imparts fruity and floral notes, and 2-undecanone, which provides floral and citrus-like aromas with peach-like nuances at low concentrations [26], were also key contributors. During fermentation, 2-nonanone was detected only in L (4.29 ± 0.23 μg/kg) and LR (7.18 ± 0.78 μg/kg), with higher levels in LR at 6 d, demonstrating that *L. plantarum* promoted its formation, while *R. mucilaginosa* further enhanced its accumulation. Similarly, 2-undecanone was absent in SF but present in L (6.67 ± 0.32 μg/kg), R (0.99 ± 0 μg/kg), and LR (8.48 ± 0.30 μg/kg), indicating that its formation was associated with microbial inoculation. The highest level was observed in LR, confirming that co-inoculation strengthened its production. Phenethyl alcohol, a compound with floral aroma, is commonly linked to yeast metabolism in fermented vegetables [27]. Its concentration increased throughout fermentation in SF, R, and LR. At 6 d, LR exhibited the highest level (7.14 ± 0.43 μg/kg, ROAV = 0.65), followed by R (3.69 ± 0.32 μg/kg, ROAV = 2.89) and SF (1.39 ± 0.08 μg/kg, ROAV = 2.3). In contrast, phenethyl alcohol was not detected in L, demonstrating that its formation depended on yeast activity and might be influenced by microbial interactions within the fermentation system.

### 2.5. Microbial Community Dynamics

#### 2.5.1. Alpha Diversity and Beta Diversity Characteristics of Microbial Communities

**Figure 5:**
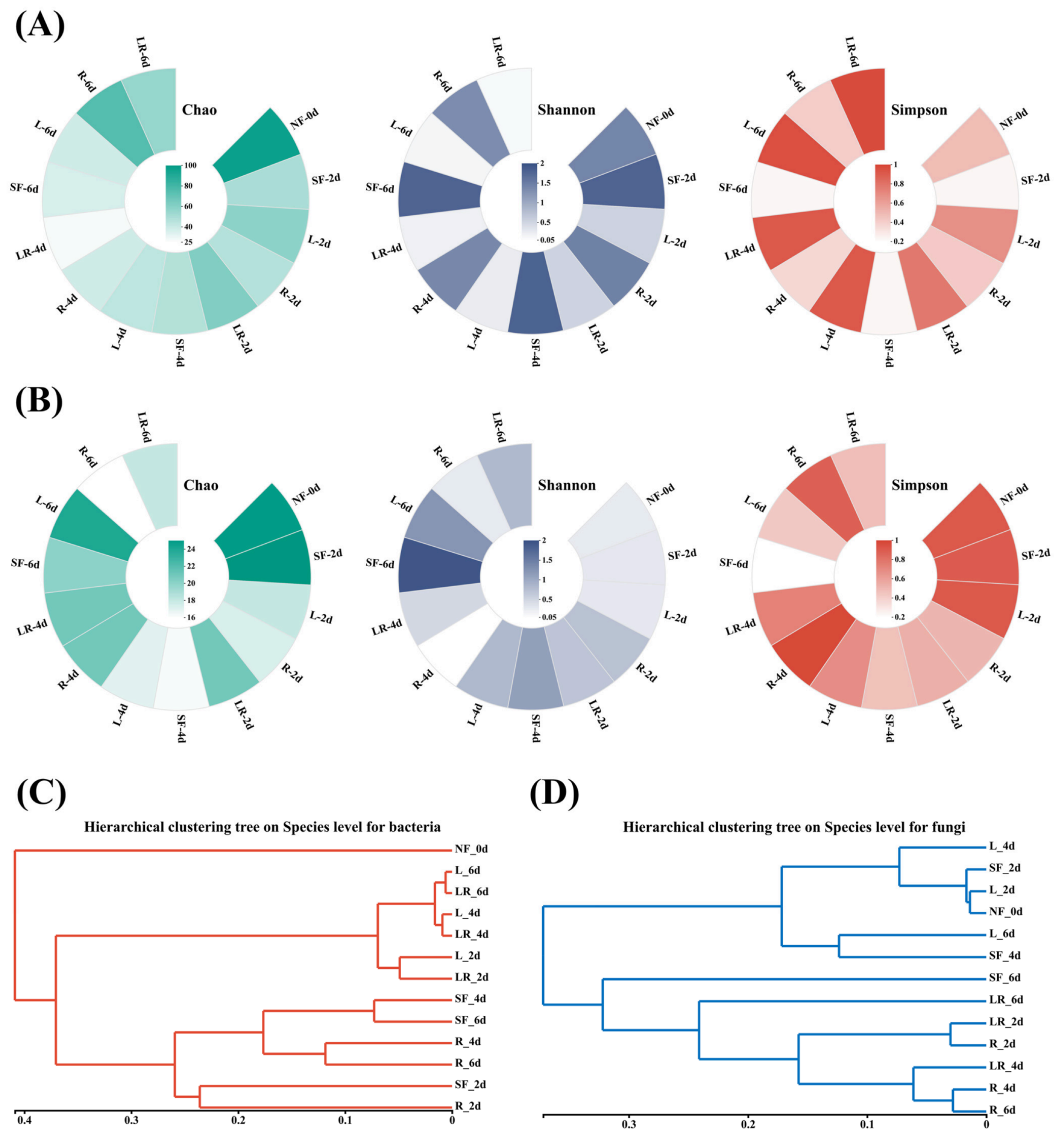
Alpha and beta diversity of microbial communities in FC samples. Chao, Shannon, and Simpson indices of (**A**) bacterial and (**B**) fungal communities; hierarchical clustering of (**C**) bacterial and (**D**) fungal beta diversity based on the Bray–Curtis distance. SF (spontaneous fermentation); L (*L. plantarum* inoculation); R (*R. mucilaginosa* inoculation); and LR (Co-inoculation of *L. plantarum* and *R. mucilaginosa* at a 1:1 ratio).

Alpha diversity was evaluated using the Chao, Shannon, and Simpson indices to characterize microbial richness, diversity, and evenness, respectively [28] (Figure 5A,B). For bacterial richness, NF (0 d) showed the highest Chao index, indicating that the raw materials contained relatively rich bacterial communities. As fermentation progressed, bacterial richness gradually decreased. However, R and LR maintained relatively higher richness on day 6 than the other groups. This result suggested that inoculation with *R. mucilaginosa* may promote bacterial growth and help maintain microbial richness during fermentation. Our previous study also demonstrated that *R. mucilaginosa* could facilitate the growth of *L. plantarum*, further supporting their synergistic interaction in co-fermentation systems [12]. For bacterial diversity, SF and R showed relatively higher Shannon indices from day 2 to day 6 than the other groups. This result suggests that free inoculation of *L. plantarum* may help maintain bacterial diversity during FC fermentation. In contrast, fungal richness and diversity showed no obvious variation among different groups throughout fermentation. This phenomenon might be attributed to the dominant role of bacteria during FC fermentation, which may suppress fungal succession and reduce fungal community variation [29].

Figure 5C,D presented the hierarchical clustering analyses of bacterial and fungal beta diversity at the species level. Branch length represents the degree of dissimilarity among samples. For bacterial communities, NF was clearly separated from the fermented groups, indicating that fermentation markedly altered the bacterial community structure. In addition, the fermented groups were divided into two clusters: (i) L and LR and (ii) R and SF. This clustering pattern was consistent with the Shannon diversity results (Figure 5A,C). The result suggested that inoculation with *L. plantarum* strongly influenced bacterial community composition. This effect remained dominant in the co-inoculation system (LR). For fungal communities, the groups were divided into two clusters: (i) L and SF and (ii) R and LR. This result indicated that *L. plantarum* exerted a relatively limited effect on fungal community composition; whereas, *R. mucilaginosa* markedly altered the fungal community structure of FC. Moreover, this effect remained dominant in the co-inoculation system. Interestingly, LR was closer to the L at the bacterial level but closer to R at the fungal level. This result implied that the LR system may simultaneously exhibit microbial characteristics driven by both *L. plantarum* and *R. mucilaginosa*, thereby contributing to a more complex flavor profile.

#### 2.5.2. Microbial Community Succession

**Figure 6:**
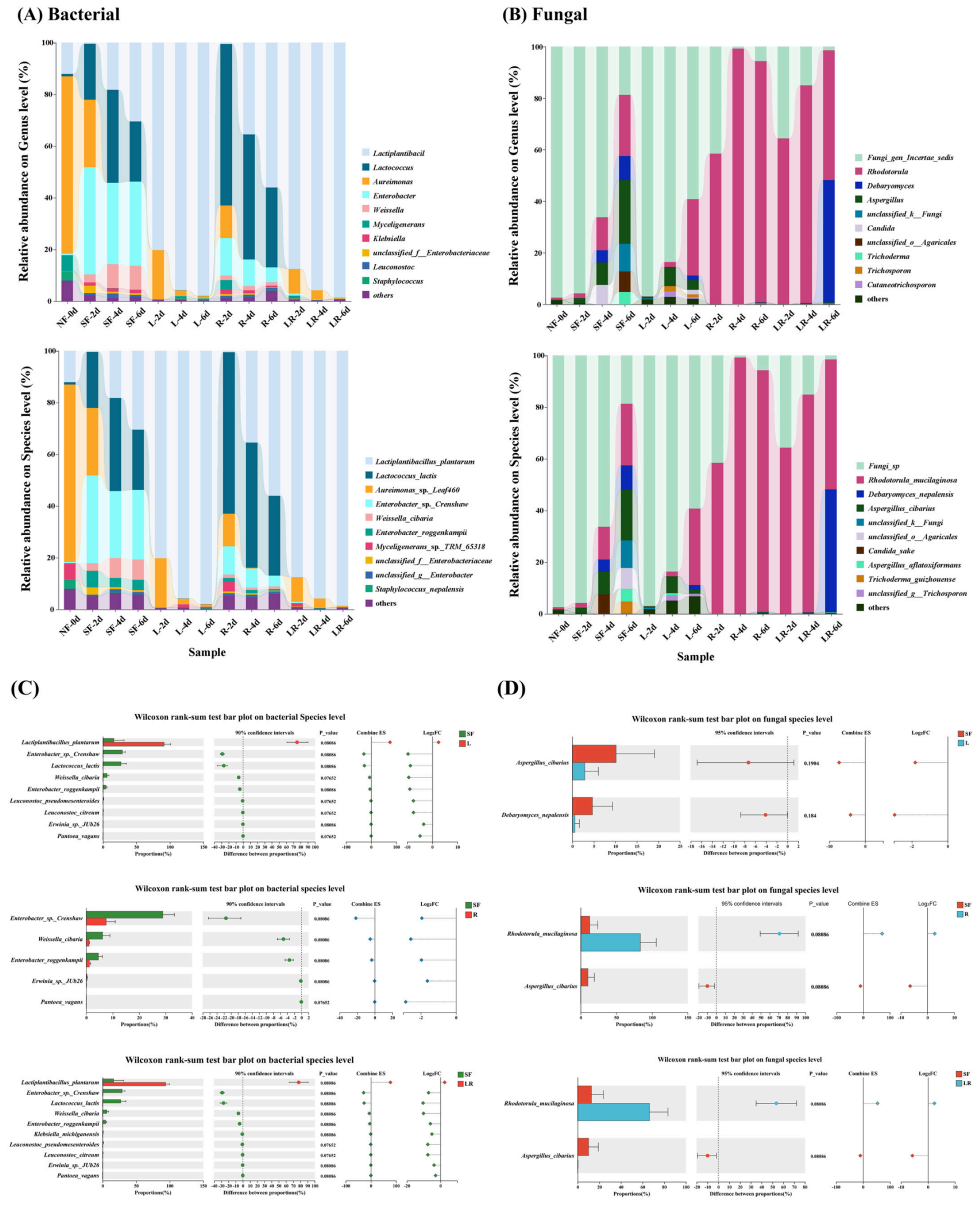
Genus/specie-level community structure and discriminatory biomarkers of FC. Dynamics of (**A**) bacterial and (**B**) fungal community composition throughout fermentation. Differential (**C**) bacterial and (**D**) fungal biomarkers identified via Wilcoxon rank-sum analysis. SF (spontaneous fermentation); L (*L. plantarum* inoculation); R (*R. mucilaginosa* inoculation); and LR (Co-inoculation of *L. plantarum* and *R. mucilaginosa* at a 1:1 ratio).

As shown in Figure 6A, the dominant bacterial genera detected in the FC raw materials and fermentation environment included *Lactiplantibacillus*, *Aureimonas*, *Myceligenerans*, and *Staphylococcus*. Among them, LAB constituted the major functional taxa during FC fermentation. The predominant LAB genera were *Lactiplantibacillus* and *Lactococcus*, mainly represented by *L. plantarum* and *Lactococcus lactis*. In L and LR, *Lactiplantibacillus* remained the dominant genus throughout fermentation, and its relative abundance gradually increased over time. In contrast, both *Lactiplantibacillus* and *Lactococcus* were dominant in SF and R, which were not inoculated with *L. plantarum*. In these groups, *Lactococcus* showed relatively high abundance during the early fermentation stage; whereas, its abundance gradually decreased as fermentation progressed. Meanwhile, the relative abundance of *Lactiplantibacillus* continuously increased. This temporal shift suggested a succession pattern between these two LAB genera. An increase in one genus was generally accompanied by a decrease in the other, which is consistent with previous studies [30]. Interestingly, the relative abundance of *Lactococcus* in R remained consistently higher than that in SF throughout fermentation, which indicated that *R. mucilaginosa* may promote the proliferation of *Lactococcus*. A possible explanation is that *R. mucilaginosa* degrades complex carbohydrates into simple sugars and secretes amino acids and other metabolites, thereby providing favorable growth conditions for LAB such as *L. plantarum* and *Lactococcus lactis* [31,32]. This interaction may facilitate LAB proliferation and contribute to the inhibition of spoilage microorganisms during fermentation [12]. For example, *Staphylococcusand* and *Enterobacter*, two species normally considered spoilage strains in foods, were also identified. However, their aboundance in SF group was significantly lower than that in the inoclated group. *R. mucilaginosa* was not the dominant microorganism in FC, it may indirectly optimize the microbial community structure by promoting LAB growth, possibly further influencing flavor development during fermentation.

The predominant fungal genera identified in the FC raw material and fermentation environment included *Rhodotorula*, *Debaryomyces*, *Aspergillus*, and *Apiotrichum* (Figure 6B). In SF, *Rhodotorula*, *Aspergillus*, *Trichoderma*, and *Debaryomyces* were the dominant fungal taxa, with the relative abundances of *Rhodotorula*, *Aspergillus*, and *Debaryomyces* increasing progressively throughout fermentation. In L, the major fungal genera included *Rhodotorula*, *Debaryomyces*, *Aspergillus*, *Candida*, and *Trichoderma*, among which *Rhodotorula* continuously increased throughout fermentation. Notably, R and LR were strongly dominated by *Rhodotorula*, whose relative abundance exceeded 50% during the middle and late fermentation stages (4–6 d). These dynamic changes indicated that inoculation with *R. mucilaginosa* substantially influenced fungal community succession and competitive interactions among fungal taxa. *Rhodotorula* exhibited strong environmental adaptability and colonization ability across all groups.

#### 2.5.3. Differential Bacterial and Fungal Species

As shown in Figure 5C,D, differential abundance analyses at the bacterial and fungal species levels were performed between the free-inoculum group (SF) and the inoculated groups (L, R, and LR) using the Wilcoxon rank-sum test. At the bacterial level, the key biomarkers distinguishing SF from L mainly included *L. plantarum*, *Enterobacter* sp. Crenshaw, *L. lactis*, *Weissella cibaria*, and *Enterobacter roggenkampii* (top five species). Similar bacterial biomarkers were also identified between SF and LR. In contrast, the top five bacterial biomarkers distinguishing SF and R were *E. Crenshaw*, *W. cibaria*, *E. roggenkampii*, *Erwinia* sp. JUb26, and *Pantoea vagans*. These differences in bacterial biomarkers suggested that inoculation with *L. plantarum* primarily contributed to the bacterial community differences observed between L/LR and SF at the species level. At the fungal level, *Aspergillus cibarius* and *Debaryomyces nepalensis* served as the major biomarkers distinguishing SF and L. In contrast, *R. mucilaginosa* and *A. cibarius* were identified as the key biomarkers distinguishing SF from R and LR. This pattern was consistent with the bacterial biomarker analysis, suggesting that inoculation with *R. mucilaginosa* mainly drove the fungal community differences between R/LR and SF at the species level. These microbial differences may further contribute to the variation in flavor profiles among different groups.

### 2.6. Correlation Analysis Among Microbial Communities During FC Fermentation

#### 2.6.1. Associations Between Key Bacterial and Fungal Communities

**Figure 7:**
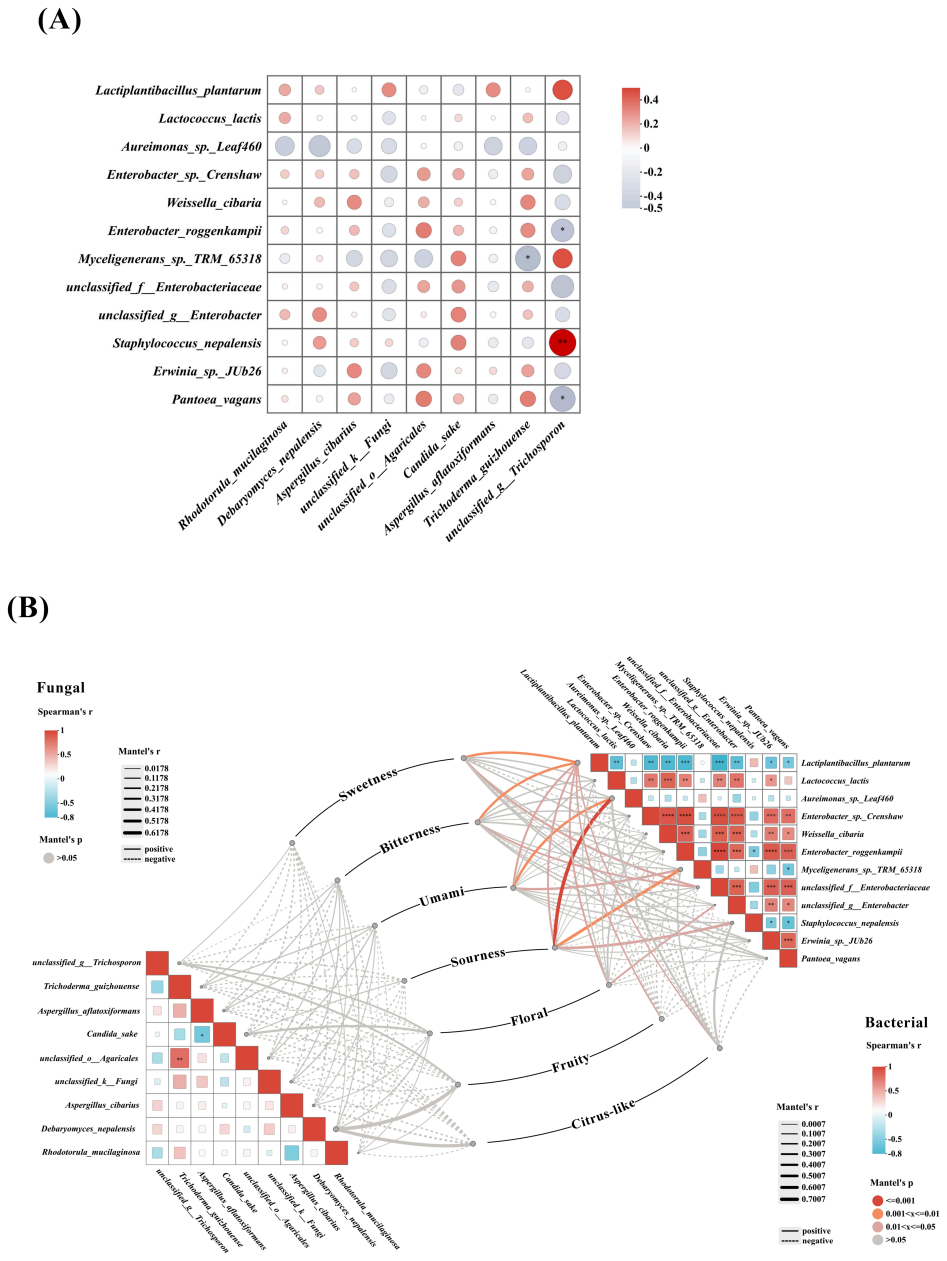
(**A**) Spearman correlation analysis of dominant fermentation microbiota at the specie level. (**B**) Mantel test analysis of key species and flavor characteristics in FC. The color gradient of Spearman’s |r| denotes the correlation strength between microbial taxa. Mantel’s r represents the correlation strength between the microbial community and flavor characteristics, with Mantel’s p indicating the statistical significance of these correlations. Asterisks indicated the significance of Spearman’s correlation: *p* < 0.05 (*), *p* < 0.01 (**), *p* < 0.001 (***), *p* < 0.0001 (****).

Spearman correlation analysis was performed to investigate the potential associations between key bacterial and fungal species in FC, including the top 10 abundant species and the top five differential species (Figure 7A). This analysis aimed to explore how *L. plantarum* and *R. mucilaginosa* interacted with other microorganisms within the FC fermentation ecosystem. Positive correlations were observed between *L. plantarum* and *R. mucilaginosa*, *D. nepalensis*, and *Aspergillus aflatoxiformans*. In contrast, *L. plantarum* showed a negative correlation with *Candida* sake. *R. mucilaginosa* exhibited positive correlations not only with *L. plantarum* but also with *L. lactis* and *Enterobacter* sp. In addition, negative correlations were observed between *R. mucilaginosa* and *Aureimonas* sp. Leaf460 as well as *Myceligenerans* sp. TRM 65318. Among these taxa, *D. nepalensis* has been reported to produce alcohols [33], which may be associated with ester-related flavor formation during fermentation, thereby potentially contributing to fruity and alcoholic aroma characteristics in FC. Its positive association with *L. plantarum* may be related to the development of desirable flavor characteristics during FC fermentation. Interestingly, *D. nepalensis* also showed a positive correlation with *Staphylococcus nepalensis*. Previous studies reported that *S. nepalensis* possesses potential beneficial functions in fermented foods and may improve the flavor quality of fermented fish sauce [34,35].

#### 2.6.2. Intra-Community Correlations Among Bacterial and Fungal Species

Spearman correlation analysis was performed among key bacterial and fungal species to explore the potential relationships within bacterial and fungal communities, respectively (Figure 7B). For bacterial species, *L. plantarum* showed significant negative correlations with most of the other key bacterial taxa, including *L. lactis*, *E. Crenshaw*, *W. cibaria*, *E. roggenkampii*, *E. JUb26*, and *P. vagans*. In contrast, *L. plantarum* was positively correlated with *S. nepalensis*. Interestingly, *Lactococcus* and *Weissella* are widely common lactic acid-producing genera in kimchi fermentation [36]. Their negative correlations with *Lactiplantibacillus* suggested potential competitive relationships among these LAB taxa. This competition may result from similar ecological functions and competition for nutrients and ecological niches during fermentation. *Enterobacter* is considered potential spoilage or harmful microorganisms in fermented foods [37]. Therefore, the presence of *L. plantarum* may contribute to the suppression of Enterobacter during FC fermentation.

Regarding the fungal community, a negative correlation was observed between *R. mucilaginosa* and *A. cibarius.* Members of the genus Aspergillus play a vital role in fermented bean products; however, they seemed to induce tissue softening in fermented vegetables [38]. Therefore, this negative correlation might potentially contribute to maintaining the textural quality of FC.

## 3. Discussion

Mantel analysis was performed to evaluate the associations between the succession of key microbial taxa and the temporal variation in flavor characteristics during FC fermentation. This analysis aimed to clarify the potential microbial contributions to flavor differences among different groups. Compared with fungal taxa, more bacterial species showed significant correlations with flavor characteristics. Among the bacterial species, *L. plantarum* exhibited positive correlations with all flavor characteristics (Mantel’s r ≤ 0.05), indicating its important role in flavor formation during FC fermentation. *L. lactis* is known to be associated with flavor development by producing sour, umami, floral, and fruity characteristics [39]. However, no significant correlations were observed between *L. lactis* and the measured flavor characteristics in this study. This result may be related to the dominance of *L. plantarum*, which potentially suppressed the metabolic contribution of *L. lactis*. This phenomenon may also explain the relatively lower flavor complexity and intensity observed in the *L. A. Leaf460* showed strong correlations with umami and sourness (Mantel’s r ≤ 0.01). *M.* TRM 65318 also showed significant correlations with umami and strong correlations with sourness (Mantel’s r ≤ 0.05). Previous studies detected *Aureimonas* during the early stages of vegetable fermentation and linked it to the formation of specific flavor compounds [40]. Although *Aureimonas* is not considered a dominant genus in fermented foods, it may function as a potential flavor-associated microorganism during vegetable fermentation. *S. nepalensis* showed a significant positive correlation with sourness (Mantel’s r ≤ 0.05). However, *S. nepalensis* is not a typical acid-producing bacterium. Therefore, this correlation may be related to its acid tolerance and coexistence with LAB, especially *L. plantarum*, under acidic fermentation conditions. Previous studies also reported that some non-LAB could indirectly participate in flavor formation during fermentation through microbial interactions and secondary metabolism [41].

Fungal taxa showed no significant correlations with flavor characteristics. This result suggests that fungi may play a relatively minor or indirect role in FC flavor formation. Therefore, during FC fermentation, *R. mucilaginosa* may function more as a community modulator by regulating the abundance of specific flavor-associated microorganisms, thereby indirectly improving the overall flavor quality of FC. Overall, *L. plantarum* acted as the primary driver of flavor formation in FC by directly shaping flavor-related bacterial succession; whereas, *R. mucilaginosa* mainly functioned as a microbial community modulator that indirectly optimized flavor quality through regulating microbial interactions and the abundance of flavor-associated taxa.

Despite the use of an OTU-based classification approach, the dominant functional taxa (e.g., *Lactiplantibacillus*, *Lactococcus*, and *Rhodotorula*) were consistently identified across all samples with high relative abundances, supporting the robustness of ecological and flavor-related interpretations.

In future studies, to further validate the effects of *L. plantarum* and *R. mucilaginosa* on the flavor of fermented cabbage, sensory evaluation, electronic nose, electronic tongue, and flavor recombination/omission experiments will be applied to further determine and distinguish how spontaneous fermentation, inoculation with either strain alone, and co-inoculation affect the presentation and perception of cabbage flavor.

## 4. Materials and Methods

### 4.1. Starter Preparation and Cabbage Fermentation

#### 4.1.1. Strain Activation and Preparation

*Lactiplantibacillus plantarum* KUST 2110 (formerly *Lactobacillus plantarum* E10) and *Rhodotorula mucilaginosa* KUST 4127 (formerly *R. mucilaginosa* E10), originally identified using the RDP Classifier (v2.2) [12] and subsequently manually verified against the current LPSN (List of Prokaryotic names with Standing in Nomenclature) database to ensure updated nomenclature. These two strains are preserved at the Yunnan Key Laboratory of Plateau Food Advanced Manufacturing (Kunming University of Science and Technology). *L. plantarum* and *R. mucilaginosa* were activated on De Man, Rogosa and Sharpe (MRS) and Yeast Extract Peptone Dextrose (YPD) agar, respectively, and incubated at 37 °C for 24 h and 30 °C for 72 h. A single colony of each strain was transferred into 100 mL of the corresponding broth and cultured under the same conditions. Cells were harvested and resuspended in sterile 4% (*w*/*v*) saline to a final concentration of 1 × 10^8^ CFU/mL, respectively. These suspensions were used as fermentation inoculation.

#### 4.1.2. Cabbage Fermentation

Fresh cabbage (*Brassica oleracea* var. *capitata* Linnaeus) was purchased from a local market located in Yuhua Street, Kunming City, Yunnan Province. Cabbage fermentation was performed following a traditional homemade procedure with minor modifications. Fresh cabbages were rinsed with tap water, air-dried at ambient temperature, cut into uniform pieces (approximately 2 cm × 6 cm), and separated into leaves and stems. Fermentation jars were sterilized with boiling water and air-dried. A 4% (*w*/*v*) brine was prepared using boiling water and cooled to room temperature (20 °C) prior to use. Equal weights of cabbage leaves and stems were placed into each jar and mixed with brine, maintaining a cabbage-to-brine ratio of 1:3 (*w*/*v*). Fermentation groups were established as follows: spontaneous fermentation (SF), *L. plantarum* inoculation (L), *R. mucilaginosa* inoculation (R), and co-inoculation of *L. plantarum* and *R. mucilaginosa* at a 1:1 ratio (LR). The total inoculum level was 2% (*v*/*v*) in all inoculated groups. After inoculation, jars were shaken for 15 s to ensure homogeneous mixing, sealed with a water lock, and incubated statically at 25 °C under aseptic conditions. Fermented cabbage (FC) samples were collected on days 0, 2, 4, and 6 using a three-point sampling method. Leaves, stems, and fermentation liquor were combined at a ratio of 1:1:3 (*w*/*w*/*v*). All samples were stored at −80 °C until analysis.

### 4.2. Determination of Physicochemical Parameters

#### 4.2.1. Total Sugars

The total sugar content in fermented cabbage was determined using a Total Sugar Content Assay Kit (Beijing Boxbio Science & Technology Co., Ltd., Beijing, China), which employs the DNS (3,5-dinitrosalicylic acid) method according to the description by Zhao et al. (2026) with some modification [42]. Briefly, 2 g homogenized sample was mixed with 8 mL of distilled water in a centrifuge tube and then subjected to ultrasonic extraction at room temperature (20 °C) for 30 min at 45 Hz. Extractions were collected by centrifugation at 3387× *g* for 5 min. An amount of 1 mL of the extract was mixed sequentially with 1 mL of 5% phenol solution and 5 mL of concentrated sulfuric acid. The mixture was gently shaken and then reacted in a boiling water bath for 10 min. After cooling for 5 min, the absorbance was measured at 490 nm. The total sugar content of the sample was calculated based on the standard curve generated from glucose standard solutions.

#### 4.2.2. pH, Titratable Acidity (TA) and Organic Acids

pH of the samples was measured using a pH meter (Mettler Toledo, Shanghai, China). Acidity was determined by using a titration device (907 Titrino, Metrohm, Herisau, Switzerland) following the method described by Shang et al. (2022) [43]. Organic acids were identified and determined as description in Yi et al. (2018) [44]. Specifically, 5 mL of the sample was mixed sequentially with 250 μL of K_4_[Fe(CN)_6_] (150 g/L) and 250 μL of ZnSO_4_ (300 g/L). After thorough mixing, the mixture was left to stand at room temperature (20 °C) for 30 min. The mixture was then centrifuged at 10,000× *g* for 20 min, and the resulting supernatant was diluted and filtered through a 0.22 μm membrane filter for analysis. Organic acids in the samples were analyzed using a HPLC system (Vanquish™ Core, Thermo Fisher Scientific, Waltham, MA, USA) equipped with a VWD. Separation was performed using a Prevail Organic Acid column (250 mm × 4.6 mm, 5 μm, Radnor, PA, USA) coupled with an Agilent 5 TC-C18 (2) guard column (12.5 mm × 4.6 mm, 5 μm, Santa Clara, CA, USA). The column oven temperature was maintained at 30 °C, and detection was carried out at 210 nm. The mobile phase consisted of 25 mM KH_2_PO_4_ (pH 2.50), delivered at a flow rate of 0.8 mL/min. Organic acids were identified by comparing retention times with those of corresponding standards and quantified using standard curves constructed from known concentrations and peak areas of the standards.

### 4.3. Free Amino Acids

The detection of amino acids was based on that reported by Liang et al. (2025) [12]. A 2 g homogenized sample was diluted to a final volume of 50 mL and allowed to stand at 4 °C for 24 h. Subsequently, 2 mL of the supernatant was mixed with 2 mL of 5% sulfosalicylic acid solution, followed by centrifugation at 6000× *g* for 10 min. The resulting supernatant was collected, and 2 mL of it was evaporated to dryness using a rotary evaporator. The residue was redissolved in 1 mL of sodium citrate buffer solution and filtered through a 0.45 μm membrane for analysis. The free amino acid content was determined using an amino acid analyzer equipped with a Na-type cation exchange chromatography column (200 mm × 4.6 mm, 8 μm) at 570 nm and 440 nm. The buffer flow rate was set at 20 mL/h, with a reaction solution flow rate of 10 mL/h. The column temperature was programmed to increase through 55 °C, 65 °C, and 77 °C, while the temperature of the ninhydrin reaction chamber was maintained at 138 °C. The injection volume was 50 μL. Amino acids were identified and quantified by comparing their retention times and peak areas with those of standard amino acid solutions.

### 4.4. Volatile Compounds

Volatile compounds were analyzed by HS-SPME-GC-MS and semi-quantified according to Wang et al. (2024) with minor modifications [15]. A total of 4 g of homogenized sample was mixed with 3 g of NaCl in a 15 mL headspace vial, followed by the addition of 20 µL hexyl acetate solution (0.1 µL/mL) as an internal standard. After equilibration at 40 °C for 15 min, volatile compounds were extracted using a 50/30 µm DVB/CAR/PDMS SPME fiber at the same temperature for 40 min. The fiber was then desorbed in the GC injection port at 250 °C for 5 min. Volatile compounds were separated and detected using a GC-MS system (7000E GC/TQ-8890 GC System, Agilent, Santa Clara, CA, USA) equipped with an HP-5MS capillary column (30 m × 0.25 mm × 0.25 μm, Agilent, Santa Clara, CA, USA). The oven temperature program was set at 45 °C for 5 min, ramped at 5 °C/min to 250 °C, and held for 2 min. Helium gas was used as the carrier at a flow rate of 2.0 mL/min. The mass spectrometer was operated with a range of 35–500 *m*/*z* under electron ionization at 70 eV, and the ion source temperature was maintained at 230 °C. Retention indices (RI) were calculated using a series of n-alkanes (C5–C25) under identical analytical conditions and were compared with reference RI values from the NIST Chemistry WebBook (https://webbook.nist.gov/chemistry/name-ser/, accessed on 10 September 2025) for compound identification.

OPLS-DA and variable importance in projection (VIP) analysis were performed to determine major volatiles responsible for the differential characteristic aroma among FC groups. Components with VIP ≥ 0.5 were recognized as differential volatile compounds, while those with VIP ≥ 1 were deemed as highly influential contributors. Additionally, relative odor activity values (ROAVs) were determined following the approach of Yang et al. (2025) [45] to screen odor-active compounds (OACs, ROAV ≥ 0.1), using the following equations:

$$OAV=\frac{C}{T},ROAV=\frac{OAVi}{OAVmax}\times 100$$

C represents the concentration of an individual compound, T denotes its odor threshold, OAV_i_ is the OAV of a given compound, and OAV_max_ corresponds to the highest OAV observed within the sample. Compounds with 1 > ROAV ≥ 0.1 were identified as potential OACs with aroma-modifying function, while compounds with ROAV ≥ 1 were recognized as key OACs.

### 4.5. High-Throughput Sequencing

#### 4.5.1. DNA Extraction, Library Preparation and Sequencing

Microbial diversity was analyzed using high-throughput sequencing by Majorbio Bio-Pharm Technology Co., Ltd. (Shanghai, China). The total DNA from samples was extracted using the E.Z.N.A.^®^ soil DNA Kit (Omega Bio-tek, Norcross, GA, USA) according to manufacturer’s instructions. DNA quality was assessed by agarose gel electrophoresis, and DNA concentration and purity were determined using a NanoDrop 2000 (Thermo Scientific, Waltham, MA, USA). The bacterial 16S rRNA were amplified using the primers 27F and 1492R, while ITS1F and ITS4R were used for the amplification of fungal ITS sequences [46,47]. After verification, purification, and quantification, purified products were used for a DNA library construction, utilizing the SMRTbell Prep Kit 3.0 (Pacific Biosciences, Menlo Park, CA, USA). The resulting SMRTbell libraries were subsequently sequenced on the PacBio Sequel IIe System (Pacific Biosciences, Menlo Park, CA, USA). High-fidelity (HiFi) reads were derived from subreads through circular consensus sequencing using the SMRT Link (version 11.0).

#### 4.5.2. Metagenomic Data Analysis

The optimized raw sequences were subjected to operational taxonomic units (OTUs) using UPARSE 7.1 with 97% sequence similarity. The most abundant sequence for each OTU was selected as a representative sequence which was analyzed by RDP Classifier (version 2.2). Alpha diversity is applied in analyzing complexity of species diversity for a sample through indices which were calculated with Mothur (version 1.30.1). Hierarchical clustering analysis of beta diversity was performed using R-3.3.1 (vegan). Community composition was analyzed using Python-2.7, and differential taxa between samples were identified using the Wilcoxon rank-sum test implemented in R-3.3.1 (stat).

The bioinformatics pipeline employed predates the current ASV-based standards and does not incorporate the latest taxonomic revisions. To ensure that the taxonomic nomenclature presented is scientifically accurate and current, all reported microbial names were updated against the LPSN (https://lpsn.dsmz.de, accessed on 10 September 2025) and the Mycobank database. All phylum, genus, and species names throughout the text were accordingly revised to conform with the most recent approved nomenclature.

### 4.6. Statistical Analysis

Experimental data were presented as mean ± standard deviation (SD). Significant differences among groups were determined using Tukey’s multiple comparison test (*p* < 0.05) in R (version 2025.05.1+513). Line charts were generated using Origin 2022. Orthogonal partial least squares discriminant analysis (OPLS-DA), variable importance in projection (VIP) calculation, and corresponding visualizations were performed using SIMCA 14.1. Bar charts were prepared on CNS platform (https://cnsknowall.com/). Spearman correlation analysis and Mantel tests were performed and generated using the Chi plot online platform (https://www.chiplot.online/) and R (version 2025.05.1+513).

## 5. Conclusions

During FC fermentation, the relationships among microbial taxa and their associations with flavor are highly complex. The inoculation of *L. plantarum* accelerates the fermentation process and simultaneously suppresses spoilage microorganisms and promotes the accumulation of flavor compounds. At the same time, the antagonistic interactions of *L. plantarum* with other LAB (e.g., *Lactococcus* and *Weissella*) and its synergistic association with *Staphylococcus* are critical for revealing the flavor-shaping patterns in FC. *R. mucilaginosa*, in turn, mainly influenced fungal community succession and indirectly affected flavor formation by regulating the abundance of flavor-associated microorganisms, including *Lactiplantibacillus* and *Lactococcus*. These findings highlighted the importance of microbial interactions in shaping the flavor characteristics of FC and provided theoretical support for improving fermented vegetable quality through targeted microbial regulation. Further exploration of the information exchange and cross-interactions among specific fermentation strains, and their effects on community succession and flavor formation, will provide deeper insights into how microbial interactions contribute to flavor determinants.

## Data Availability

Data will be made available on request.

## Supplementary Materials

The following supporting information can be downloaded at: https://www.mdpi.com/article/10.3390/molecules31152666/s1, Table S1 The content of organic acids in different groups of FC ^a^; Table S2 The content of free amino acids in different groups of FC ^a^; Table S3 Concentration of volatile flavor substances in different fermentation groups ^a^; Table S4 ROAV of volatile flavor substances in different fermentation groups ^a^. Refs. [48,49,50,51,52,53,54,55,56,57,58] are cited in Supplementary Materials.

## Abbreviations

The following abbreviations are used in this manuscript:

FC	Fermented cabbage
LAB	Lactic acid bacteria
SF	Spontaneous fermentation
L	*L. plantarum* inoculation
R	*R. mucilaginosa* inoculation
LR	Co-inoculation of *L. plantarum* and *R. mucilaginosa* at a 1:1 ratio
SD	Standard deviation
FAA	Free amino acid
MRS	De Man, Rogosa and Sharpe
YPD	Yeast Extract Peptone Dextrose
