# Supplementary material for: Microbial Interactions and Flavor Modulation in Cabbage Fermentation: Roles of Lactiplantibacillus plantarum and Rhodotorula mucilaginosa
Source: Molecules. 2026 Jul 30;31(15):2666. doi: 10.3390/molecules31152666 (PMC13468091; doi:10.3390/molecules31152666)
Supplement: Supplementary file 1 [file molecules-31-02666-s001.zip › molecules-4395332-supplementary.pdf]

Supplementary data

Table S1 The content of organic acids in different groups of FC[a]

Organic acids	Concentration (mg/g)												
	0d	2d				4d				6d			
	NF	SF	L	R	LR	SF	L	R	LR	SF	L	R	LR
Oxalic acid	4.52±0	2.53±0^{b}	4.73±0.02^{a}	4.06±0^{b}	3.58±0^{c}	0.69±0^{D}	1.77±0^{B}	1.50±0^{C}	1.91±0^{A}	0.47±0^{iiii}	1.78±0^{ii}	1.48±0^{iii}	1.82±0^{i}
Tartaric acid	0.05±0	0.06±0^{a}	0.01±0^{c}	0.05±0^{b}	-	-	-	-	-	-	-	-	-
Quininic acid	1.91±0.06	1.39±0^{a}	1.06±0.03^{c}	1.24±0.02^{b}	0.64±0.03^{d}	-	-	0.34±0.01^{A}	-	-	-	-	-
Malic acid	0.28±0	0.59±0.01^{a}	0.17±0^{d}	0.49±0.01^{b}	0.22±0.01^{c}	0.22±0^{A}	0.02±0^{B}	-^{C}	0.01±0^{BC}	-	-	-	-
Lactic acid	0.11±0.01	0.50±0^{d}	1.80±0.01^{b}	0.58±0.01^{c}	1.87±0.01^{a}	1.51±0^{C}	2.93±0.02^{A}	2.10±0.02^{B}	2.93±0.01^{A}	2.79±0.02^{iiii}	3.44±0^{ii}	3.53±0.01^{i}	3.24±0.03^{iii}
Aacetic acid	-	0.09±0^{a}	0.03±0^{c}	0.07±0^{b}	0.02±0^{d}	0.31±0^{A}	0.21±0^{C}	0.17±0^{D}	0.24±0^{B}	0.25±0.01^{i}	0.22±0^{ii}	0.19±0^{iii}	0.21±0^{ii}
Citric acid	0.38±0.01	0.72±0.01^{a}	0.61±0^{b}	0.72±0.01^{a}	0.53±0^{c}	0.50±0^{A}	0.23±0^{B}	0.49±0^{A}	0.25±0.01^{B}	0.22±0^{iii}	0.28±0.01^{ii}	0.49±0^{i}	0.26±0.01^{ii}
Succinic acid	0.92±0.08	-	-	-	-	-	-	-	-	-	-	-	-
Fumaric acid	-	0.02±0^{a}	-	0.02±0^{b}	0.01±0^{c}	-	-	-	-	-	-	-	-

[a] "-" indicates not detected; Different letters at the same fermentation stage indicated significant differences in the Tukey test (p<0.05).

Table S2 The content of free amino acids in different groups of FC[a]

Free amino acids	Concentration (mg/kg)												
	0d	2d				4d				6d			
	NF	SF	L	R	LR	SF	L	R	LR	SF	L	R	LR
Asp	102.00±0.81	101.00±0.81^{b}	62.00±0.81^{c}	180.00±1.63^{a}	58.50±0.40^{d}	42.00±0^{A}	25.00±0^{C}	43.00±0^{A}	27.00±0.81^{B}	25.00±0^{k}	26.00±0^{j}	35.50±0.40^{i}	21.50±0.40^{l}
Thr	34.50±1.22	34.00±0.81^{b}	26.50±0.40^{c}	42.50±1.22^{a}	23.50±1.22^{c}	18.50±0.40^{A}	8.50±0.40^{D}	16.00±0^{B}	10.50±0.40^{C}	12.00±0^{i}	9.00±0.81^{j}	12.50±0.40^{i}	9.00±0^{j}
Ser	609.00±0	684.50±2.04^{a}	541.00±0.81^{b}	546.50±3.67^{b}	424.00±2.44^{c}	247.50±1.22^{A}	164.50±0.40^{C}	183.50±0.40^{B}	182.00±0.81^{B}	155.50±0.40^{i}	137.00±0.81^{j}	153.50±0.40^{i}	116.50±1.22^{k}
Glu	154.50±1.22	132.50±1.22^{b}	104.00±0^{c}	144.50±2.85^{a}	94.00±0^{d}	42.50±0.4^{B}	39.00±0^{C}	35.00±0^{D}	46.00±0^{A}	20.00±0^{l}	39.50±0.40^{i}	32.00±0^{k}	38.50±0.40^{j}
Gly	8.50±0.40	14.50±0.40^{b}	11.00±0^{c}	19.50±0.40^{a}	9.50±0.40^{d}	9.00±0.81^{AB}	5.00±0.81^{C}	10.00±1.41^{A}	6.00±0.81^{BC}	7.00±0^{j}	5.00±0^{k}	11.00±0^{i}	5.50±0.40^{k}
Ala	61.50±0.40	141.50±0.40^{b}	99.50±0.40^{d}	152.00±0.81^{a}	109.00±0.81^{c}	79.00±0.81^{A}	40.00±2.94^{D}	61.00±1.41^{B}	51.00±0.81^{C}	58.00±0^{i}	43.00±0.81^{k}	56.00±0^{j}	40.00±0^{l}
Cys	27.5±0.4	29±0	25±0	28±0	24±0	25.5±0.4^{A}	23±0^{C}	24±0^{B}	23±0^{C}	23.5±0.4^{ij}	23±0^{k}	24±0^{i}	23±0^{j}
Val	43.50±0.40	80.50±0.40^{b}	57.50±0.40^{c}	95.00±0.81^{a}	50.50±0.40^{d}	35.50±0.40^{A}	20.00±0^{D}	33.00±0^{B}	22.00±0^{C}	25.50±0.40^{j}	19.50±0.40^{k}	29.00±0^{i}	17.50±0.40^{l}
Met	5.00±0	11.00±0.81^{b}	9.00±0.08^{c}	13.00±0.81^{a}	7.00±0.21^{d}	5.00±0^{B}	4.00±0^{C}	6.00±0^{A}	4.50±0.40^{BC}	3.00±0^{l}	3.50±0.40^{k}	5.50±1.22^{i}	4.00±0^{j}
Ile	30.50±0.40	54.00±0^{b}	34.50±0.40^{c}	62.00±0^{a}	30.00±0^{d}	20.50±0.40^{A}	10.00±0^{C}	21.00±0^{A}	12.00±0^{B}	12.00±0^{j}	12.00±0.81^{j}	16.00±0.81^{i}	9.00±0^{k}
Leu	31.50±0.40	64.00±0^{b}	49.50±1.22^{c}	75.50±2.04^{a}	38.00±0^{d}	28.00±0.81^{A}	14.00±0.21^{B}	27.00±1.63^{A}	14.00±0.81^{B}	15.50±0.40^{j}	14.50±0.40^{j}	21.00±0^{i}	10.50±0.40^{k}
Tyr	20.00±0	44.50±0.40^{b}	30.00±0.81^{c}	52.00±0^{a}	23.50±0.40^{d}	21.00±0^{A}	4.00±0^{D}	18.50±0.40^{B}	6.50±0.40^{C}	7.00±0^{i}	8.00±1.63^{i}	10.00±1.63^{i}	2.50±0.40^{j}
Phe	34.00±0	62.50±0.	50.50±0.	77.00±0^{a}	37.50±0.	27.00±0^{A}	7.00±0^{D}	24.50±0.	9.00±0^{C}	11.00±0^{j}	7.50±0.4	15.50±0.	4.50±0.4

		40^{b}	40^{c}		40^{d}			40^{B}			0^{k}	40^{i}	0^{l}
His	47.00±0	59.00±0^{a}	51.00±0^{c}	55.50±0.40^{b}	31.00±0^{d}	21.00±0.81^{A}	17.00±0.81^{B}	22.00±1.41^{A}	19.00±0.81^{AB}	15.00±0^{k}	16.00±0^{j}	20.50±0.40^{i}	15.00±0^{k}
Lys	37.50±0.40	193.50±1.22^{a}	46.50±0.40^{c}	86.50±4.49^{b}	31.50±0.40^{d}	42.00±0^{B}	11.50±0.40^{D}	48.00±0^{A}	13.00±0^{C}	19.00±0^{j}	11.00±0^{k}	41.50±1.22^{i}	9.50±0.40^{k}
Arg	184.50±0.40	48.00±1.63^{d}	187.00±0^{a}	102.00±0.81^{c}	164.50±1.22^{b}	19.00±0.81^{C}	72.00±0.81^{B}	20.00±0^{C}	74.50±0.40^{A}	1.00±0^{l}	63.50±0.40^{i}	4.50±0.40^{k}	56.00±0^{j}
Pro	12.00±0	53.00±0.81^{a}	28.50±0.40^{c}	42.50±0.40^{b}	18.00±0^{d}	12.50±0.4^{B}	10.50±0.40^{C}	14.00±0^{A}	11.50±0.40^{BC}	13.00±0^{k}	15.50±0.40^{j}	17.50±0.40^{i}	8.50±0.40^{l}
Sweet amino acids	806.5±0.5	1201.5±5.5^{a}	810.5±0.5^{c}	984.5±6.5^{b}	666±3^{d}	444±1.73^{A}	260±4.35^{D}	365.5±1.32^{B}	296±1^{C}	290±1^{j}	240±3^{k}	321±3^{i}	206.5±0.5^{l}
Bitter amino acids	380±0	372±2.64^{c}	436.5±3.55^{b}	465±3^{a}	355.5±1.31^{d}	167±1.73^{A}	151±1.99^{C}	163±1^{B}	162.5±0.86^{B}	88±0^{l}	148±4^{i}	117±2^{k}	124.5±0.5^{j}
Umami amino acids	256.5±0.5	233.5±2.5^{b}	166±1^{c}	324.5±5.5^{a}	152.5±0.5^{d}	84.5±0.5^{A}	64±0^{D}	78±0^{B}	73±1^{C}	45±0^{l}	65.5±0.5^{j}	67.5±0.5^{i}	60±0^{k}
Total amino acids	1443±1	1807±1^{a}	1413±2.05^{c}	1774±2^{b}	1174±2.2^{d}	695.5±2.78^{A}	475±3.5^{D}	606.5±1.8^{B}	531.5±0.5^{C}	423±1^{k}	453.5±1.5^{j}	505.5±1.5^{i}	391±0^{l}

[a] Different letters at the same fermentation stage indicated significant differences in the Tukey test (p<0.05)

Table S3 Concentration of volatile flavor substances in different fermentation groups[a]

Compounds	CAS NO.	RI	RI*	Concentration (μg/kg)												
				0d	2d				4d				6d			
				NF	SF	L	R	LR	SF	L	R	LR	SF	L	R	LR
**Alcohols**																
2-Ethyl-1-hexanol	104-76-7	1035.17	1034	2.60±0.43	ND	ND	ND	ND	1.07±0.13	1.60±0.03	1.05±0.50	ND	0.88±0.12	ND	0.87±0.19	ND
1-Hexanol	111-27-3	874.17	870	155.46±9.42	101.17±7.11	39.84±4.05	202.66±3.31	ND	ND	ND	ND	ND	ND	ND	ND	ND
1-Octen-3-ol	3391-86-4	984.95	986	3.27±0.67	4.64±1.44	141.92±19.54	15.69±4.24	110.57±26.64	ND	52.93±3.79	ND	52.87±11.09	40.44±5.29	53.29±10.94	58.46±6.50	15.71±1.12
1-Nonanol	143-08-8	1175.22	1175	ND	ND	12.73±1.53	0.94±0.13	11.11±0.65	ND	2.74±0.31	ND	3.13±0.62	0.90±0.02	5.68±0.22	ND	5.11±0.59
2-Nonanol	628-99-9	1102.58	1098	ND	ND	ND	ND	ND	ND	1.51±0.07	ND	1.23±0.25	ND	2.87±0.34	1.02±0.03	5.14±0.39
2-Undecanol	1653-30-1	1301.8	1303	ND	ND	ND	ND	ND	ND	1.91±0.18	ND	2.08±0.51	1.09±0.43	4.28±0.28	ND	8.15±1.33
Phenylethyl alcohol	60-12-8	1112.79	1112	ND	0.13±0	ND	0.53±0.08	0.33±0.02	1.79±0.06	ND	2.87±0.17	2.24±0.24	1.39±0.08	ND	3.69±0.32	7.14±0.43
3-Methyl-1-butanol	123-51-3	733.46	734	ND	ND	ND	ND	ND	24.39±1.37	ND	37.32±1.28	ND	31.36±2.13	ND	47.13±3.45	ND
2,3-Butanediol	513-85-9	790.13	793	ND	ND	ND	ND	ND	22.44±1.05	ND	ND	ND	30.2±2.06	ND	ND	ND
(Z)-2-Penten-1-ol	1576-95-0	768.54	769	ND	16.82±3.04	ND	31.43±6.18	ND	2.89±1.64	ND	ND	ND	ND	ND	ND	ND
(Z)-3-Hexen-1-ol	928-96-1	859.37	859	ND	ND	ND	ND	ND	0.97±0.09	ND	1.28±0.23	ND	ND	ND	ND	ND

**Terpenes**																
Eucalyptol	470-82-6	1032.95	1033	1.03±0.07	0.49±0.02	ND	ND	ND	0.16±0.01	ND	0.27±0.01	ND	0.2±0.01	ND	0.54±0.04	ND
Linalool	78-70-6	1100.74	1100	ND	ND	ND	ND	ND	ND	0.95±0.01	ND	1.15±0.02	ND	1.45±0.07	0.87±0.08	2.24±0.10
Geraniol	106-24-1	1257.47	1257	ND	ND	ND	ND	ND	ND	1.96±0.25	ND	1.43±0.34	1.08±0.39	2.67±0.54	ND	4.33±0.53
Menthol	89-78-1	1173.54	1173	ND	0.11±0.03	ND	0.24±0.09	ND	ND	ND	ND	ND	ND	ND	ND	ND
Carvacrol	499-75-2	1312.98	1312	ND	ND	ND	0.32±0.05	ND	ND	ND	ND	ND	ND	ND	ND	ND
**Phenols**																
2,6-Di-tert-butylphenol	96-76-4	1512.71	1513	ND	1.41±0.10	1.23±0.01	1.10±0.14	1.08±0	0.26±0.01	1.69±0.05	ND	1.42±0.23	0.42±0.06	0.77±0.06	0.87±0.06	1.15±0.02
**Ketones**																
Isophorone	78-59-1	1120.3	1118	0.81±0.18	0.09±0.03	0.18±0.05	0.19±0.03	0.11±0.04	0.15±0.07	0.18±0.03	0.25±0.09	0.21±0	0.18±0.05	0.16±0.02	0.32±0.07	0.43±0.08
β-Lonone	79-77-6	1486.13	1486	0.84±0.02	0.34±0.02	0.65±0	0.55±0.04	0.61±0.07	0.16±0.01	0.98±0.09	0.16±0	0.93±0.12	0.07±0.01	ND	0.14±0.01	ND
6-methyl-5-hepten-2-one	110-93-0	991.02	991	ND	ND	22.69±2.03	ND	12.99±2.09	ND	22.31±0.65	ND	19.19±0.82	2.87±0.37	11.34±0.88	8.55±0.29	ND
2-Tridecanone	593-08-8	1495.94	1494	ND	0.05±0.01	0.11±0.02	0.04±0	0.04±0	ND	1.19±0.17	0.26±0.15	1.13±0.24	0.37±0.27	1.97±0.28	0.70±0.13	2.83±0.49
2-Nonanone	821-55-6	1094.25	1095	ND	ND	ND	ND	ND	ND	5.79±0.35	ND	3.05±0.34	ND	4.29±0.23	ND	7.18±0.78
2-Undecanone	112-12-9	1294.53	1293	ND	ND	ND	ND	ND	ND	6.58±0.08	0.28±0	5.34±0.23	ND	6.67±0.32	0.99±0	8.48±0.30
β-Damascenone	23726-93-4	1384.66	1386	ND	ND	ND	ND	ND	ND	0.09±0	ND	ND	ND	0.15±0.02	ND	ND

3-Octanone	106-68-3	990.58	990	ND	ND	ND	ND	ND	ND	ND	ND	ND	ND	ND	ND	46.93±5.21
**Aldehydes**																
(E,E)-2,4-Heptadienal	4313-03-5	998.04	1007	2.55±0.26	2.38±0.16	34.52±1.94	7.06±0.13	11.63±0.82	3.04±0.23	14.14±1.11	2.61±0.20	24.33±1.40	3.94±0	25.22±1.38	5.80±0.56	51.12±2.35
(E,E)-2,4-Nonadienal	5910-87-2	1214.23	1214	0.67±0	0.58±0.06	3.03±0.10	1.46±0.10	3.08±0.17	ND	1.22±0.14	ND	2.14±0.25	ND	3.01±0.18	0.74±0.05	5.07±0.22
(E)-2-Decenal	3913-81-3	1263.68	1261	ND	ND	11.40±0.93	1.39±0.23	10.59±1.98	ND	6.50±0.99	1.21±0.54	7.80±1.77	1.63±0.41	9.20±0.46	2.55±0.38	16.00±1.76
(E)-2-Heptenal	18829-55-5	963.23	962	8.45±0.79	11.96±1.07	109.49±4.15	25.62±2.39	61.51±4.11	16.86±0.45	44.43±0.91	21.15±1.59	103.12±2.10	55.80±1.73	91.98±5.90	69.00±1.53	187.57±7.71
(E)-2-Hexenal	6728-26-3	856.08	855	272.68±12.48	297.17±5.98	84.80±1.69	534.13±6.48	38.96±1.98	ND	4.43±0.20	ND	7.60±0.77	2.19±0.03	7.46±0.26	ND	17.63±0.64
(E)-2-Nonenal	18829-56-6	1163.15	1163	0.48±0.07	1.92±0.24	2.90±0.84	4.92±1.48	2.15±0.52	ND	1.42±0.20	ND	2.50±0.58	0.22±0.05	3.43±0.24	0.44±0.14	5.58±0.61
(E)-2-Octenal	2548-87-0	1062.7	1063	5.73±0.14	1.43±0.21	0.84±0.03	6.11±0.14	23.51±0.27	ND	21.43±0.52	ND	38.91±0.88	4.51±0.28	1.5±0.10	7.51±0.58	86.84±3.69
Benzeneacetaldehyde	122-78-1	1046.66	1046	2.96±0.55	1.32±0.23	ND	ND	ND	1.05±0.22	ND	ND	ND	ND	ND	ND	ND
n-Decanal	112-31-2	1206.97	1206	1.02±0.08	0.77±0.08	2.11±0.57	1.19±0.14	3.09±0.36	0.67±0.09	1.72±0.22	0.50±0.26	2.65±0.47	1.12±0.64	4.18±0.80	0.57±0.13	4.81±1.15
Hexanal	66-25-1	802.06	800	553.09±7.15	96.56±3.14	67.25±0.85	66.96±2.45	85.32±0.14	ND	56.97±2.76	ND	126.82±10.02	ND	110.80±4.43	ND	225.26±2.95
Neral	106-26-3	1272.75	1270	2.19±2.06	0.63±0.08	ND	ND	ND	ND	1.49±0.25	ND	ND	ND	3.36±0.98	ND	ND
Nonanal	124-19-6	1105.85	1105.9	6.16±0.56	14.1±0.27	13.53±0.48	13.85±1.75	18.09±2.45	10.09±0.83	10.58±0.21	9.15±2.56	13.96±0.92	3.05±1.36	13.53±1.50	6.95±1.91	23.86±1.56
(E)-2-Pentenal	1576-87-0	754.24	754	ND	ND	0.69±0.43	ND	4.49±0.49	ND	11.67±0.94	ND	13.45±0.15	ND	11.76±0.70	ND	26.86±1.94
(E)-2-Undecenal	53448-07-0	1365.05	1364	ND	0.24±0.02	7.24±0.51	0.7±0.05	7.47±0.22	ND	3.64±0.13	ND	4.59±0.47	1.56±0.12	8.82±0.45	1.88±0.09	12.61±0.72

Octanal	124-13-0	1004.62	1004	ND	ND	ND	ND	ND	ND	7.68±0.32	ND	11.76±0.60	ND	16.45±0.40	ND	32.24±1.61
2,6-(E,Z)-Nonadienal	557-48-2	1156.33	1156	ND	2.74±0.07	ND	7.17±0.33	0.60±0.01	ND	ND	ND	1.13±0.09	0.30±0	2.60±0.12	ND	3.15±0.13
(E,E)-2,4-Decadienal	25152-84-5	1316.41	1318	ND	ND	ND	0.71±0.06	1.72±0.10	ND	ND	0.40±0.01	ND	ND	ND	1.46±0.03	ND
2,4-Decadienal	2363-88-4	1316.49	1317	ND	ND	ND	ND	ND	ND	ND	ND	1.51±0.23	ND	ND	ND	6.77±1.58
**Acids**																
Octanoic acid	124-07-2	1179.48	1180	ND	ND	ND	ND	ND	1.23±0.15	1.22±0.07	1.85±0.17	2.78±0.56	2.24±0.54	2.64±0.41	1.92±0.28	ND
Nonanoic acid	112-05-0	1272.76	1272.1	ND	ND	ND	ND	ND	0.87±0.27	ND	ND	ND	1.51±0.4	ND	1.63±0.28	ND
**Esters**																
Methyl nonanoate	1731-84-6	1228.16	1227	0.09±0.01	0.11±0	ND	0.17±0.01	ND	ND	ND	ND	ND	ND	ND	ND	ND
Ethyl 3-phenylpropionate	2021-28-5	1350.68	1347	ND	ND	0.55±0.06	0.21±0.06	0.75±0.13	ND	0.22±0.02	0.21±0.09	ND	ND	ND	ND	ND
Ethyl caprylate	106-32-1	1200.25	1199	ND	ND	2.41±0.98	ND	ND	ND	ND	ND	ND	ND	ND	ND	ND
γ-Undecalactone	104-67-6	1571.1	1573	0.05±0	ND	0.01±0	ND	ND	ND	0.05±0	ND	0.06±0	ND	0.16±0	0.03±0	ND
**Sulfides**																
Allyl isothiocyanate	57-06-7	886.88	890	ND	6.85±0.76	63.64±0.34	29.91±0.42	309.04±17.69	325.68±12.82	117.73±2.56	387.01±0.56	101.54±6.58	6.68±0.45	132.23±8.11	33.19±0.61	34.17±3.64
2-Isothiocyanatobutane	4426-79-3	938.87	933.3	ND	ND	ND	1.75±0.27	3.69±0.37	1.77±0.42	0.50±0.30	1.25±0.60	0.68±0.25	ND	ND	0.64±0.31	ND
1-Isothiocyanatobut	592-82-5	959.32	959	ND	ND	ND	ND	ND	0.57±0.19	0.48±0.14	0.67±0.17	ND	ND	0.45±0.04	0.31±0.19	ND

ane																
3-Methylthiopropyl isothiocyanate	505-79-3	1310.63	1316.1	ND	ND	ND	ND	ND	7.50±1.97	4.52±0.89	3.17±3.34	1.55±1.06	ND	3.10±0.70	0.03±0.01	ND
Phenethyl isothiocyanate	2257-09-2	1465.38	1465	ND	ND	ND	ND	1.22±0.48	33.36±7.31	5.91±0.72	11.96±1.34	3.09±0.12	ND	6.27±0.76	0.47±0.16	3.27±0.36
3-Butenyl isothiocyanate	3386-97-8	986.57	982.6	ND	ND	ND	ND	ND	22.65±2.08	ND	3.80±0.70	ND	ND	ND	ND	ND
Benzyl isothiocyanate	622-78-6	1364.44	1359	ND	ND	ND	ND	ND	1.46±0.10	ND	1.45±0.13	ND	ND	ND	ND	ND
Erucin	4430-36-8	1432.91	1432	ND	ND	ND	ND	ND	0.11±0.01	ND	ND	ND	ND	ND	ND	ND
**Others**																
5-Methylthiopentanonitrile	59121-25-4	1200.75	1202.3	6.54±0.58	5.85±0.70	ND	15.64±2.42	5.68±0.90	2.27±0.36	ND	ND	1.6±0.25	ND	ND	0.64±0.07	4.92±0.95
Benzenepropanenitrile	645-59-0	1241.45	1242.6	42.7±0.17	45.25±2.53	33.00±1.24	62.72±5.31	24.05±1.26	3.28±0.28	2.60±0.10	1.32±0.09	2.43±0.2	3.06±0.26	3.8±0.33	1.90±0.14	5.33±0.44
Benzeneacetonitrile	140-29-4	1140.8	1140	3.39±0.65	17.40±2.67	13.99±0.55	31.26±7.57	9.29±1.79	1.12±0.13	1.14±0.09	1.29±0.73	1.19±0.57	0.79±0.18	ND	1.03±0.10	1.68±0.22

[a] ND indicates that the substance was not detected, RI is the retention index calculated for the substance, and RI* is the reference retention index of the substance in relevant literature. The reference retention index is from the National Institute Standards and Technology Chemistry WebBook, SRD 69 (NIST) (https://webbook.nist.gov/chemistry/name-ser/)

Table S4 ROAV of volatile flavor substances in different fermentation groups[a]

Compounds	CAS	Thershold (μg/kg)	Description	ROAV												
				0d	2d				4d				6d			
				NF	SF	L	R	LR	SF	L	R	LR	SF	L	R	LR
**Alcohols**																
2-Ethyl-1-hexanol	104-76-7	0.3	ethereal, fruity	3.76	0	0	0	0	35.57	0.56	8.53	0	4.38	0	2.05	0
1-Hexanol	111-27-3	590	green, grassy	0.11	0.02	0.04	0.02	0	0	0	0	0	0	0	0	0
1-Octen-3-ol	3391-86-4	1[A]	mushroom-like	1.41	0.75	100	0.98	37.61	0	5.62	0	6.92	60.17	5.58	41.18	1.29
1-Nonanol	143-08-8	NF	soapy, fruity	-	-	-	-	-	-	-	-	-	-	-	-	-
2-Nonanol	628-99-9	NF	fruity, green	-	-	-	-	-	-	-	-	-	-	-	-	-
2-Undecanol	1653-30-1	NF	soapy	-	-	-	-	-	-	-	-	-	-	-	-	-
Phenylethyl Alcohol	60-12-8	0.9	floral, honey-like	0	0.02	0	0.03	0.12	19.79	0	7.75	0.32	2.3	0	2.89	0.65
3-Methyl-1-butanol	123-51-3	220	malty	0	0	0	0	0	1.09	0	0.41	0	0.21	0	0.15	0
2,3-Butanediol	513-85-9	95.1[B]	butter-like, sweet	0	0	0	0	0	2.33	0	0	0	0.47	0	0	0
(Z)-2-Penten-1-ol	1576-95-0	NF	fruity	-	-	-	-	-	-	-	-	-	-	-	-	-
(Z)-3-Hexen-1-ol	928-96-1	3.9	lettuce-like	0	0	0	0	0	2.47	0	0.8	0	0	0	0	0
**Terpenes**																
Eucalyptol	470-82-6	12[C]	peppermint-like	0.03	0	0	0	0	0.13	0	0.05	0	0.02	0	0.03	0

Linalool	78-70-6	0.087	citrus-like, floral	0	0	0	0	0	0	1.16	0	1.74	0	1.75	7.12	2.12
Geraniol	106-24-1	1.1	flowery, rose-like	0	0	0	0	0	0	0.19	0	0.17	1.47	0.25	0	0.32
Menthol	89-78-1	NF	mint-like	-	-	-	-	-	-	-	-	-	-	-	-	-
Carvacrol	499-75-2	0.033	phenolic, leather-like	0	0	0	0.61	0	0	0	0	0	0	0	0	0
**Phenols**																
2,6-Di-tert-butylphenol	96-76-4	NF	phenolic-like, leather-like	-	-	-	-	-	-	-	-	-	-	-	-	-
**Ketones**																
Isophorone	78-59-1	200[D]	Cedarwood, Spice	0	0	0	0	0	0	0	0	0	0	0	0	0
β-Lonone	79-77-6	0.021	flowery, violet-like	17.41	2.67	21.87	1.65	9.92	80.07	4.98	19.09	5.84	5.66	0	4.97	0
6-methyl-5-Hepten-2-one	110-93-0	18.89[E]	Citrus, Mushroom, Pepper, Rubber, Strawberry	0	0	0.84	0	0.23	0	0.12	0	0.13	0.22	0.06	0.31	0
2-Tridecanone	593-08-8	NF	rancid, fruity, tallow-like	-	-	-	-	-	-	-	-	-	-	-	-	-
2-Nonanone	821-55-6	5[A]	fruity, flowery, fatty	0	0	0	0	0	0	0.12	0	0.07	0	0.09	0	0.11
2-Undecanone	112-12-9	0.007[F]	fruity, rose-like, orange-like	0	0	0	0	0	0	100	100	100	0	100	100	100
β-Damascenone	23726-93-4	0.006	cooked apple-like,fruity, sweet, honey-like	0	0	0	0	0	0	1.75	0	0	0	2.76	0	0
3-Octanone	106-68-3	28[G]	citrus-like, fruity	0	0	0	0	0	0	0	0	0	0	0	0	0.13
**Aldehydes**																
(E,E)-2,4-Heptadienal	4313-03-5	NF	fatty, flowery	-	-	-	-	-	-	-	-	-	-	-	-	-
(E,E)-2,4-Nonadienal	5910-87-2	0.046	fatty, soapy	6.36	2.06	46.56	1.99	22.84	0	2.84	0	6.11	0	6.88	11.33	9.11
(E)-2-Decenal	3913-81-3	0.3[H]	fatty, green	0	0	26.77	0.29	12.01	0	2.3	9.81	3.4	8.08	3.21	6	4.4
(E)-2-Heptenal	18829-55-5	NF	oily, fatty, window putty-like	-	-	-	-	-	-	-	-	-	-	-	-	-

(E)-2-Hexenal	6728-26-3	110	oily, fatty, window putty-like, deep-fried	1.07	0.44	0.54	0.3	0.12	0	0	0	0	0.02	0	0	0.01
(E)-2-Nonenal	18829-56-6	0.19	tallow-like, green leaf-like	1.11	1.65	10.76	1.62	3.86	0	0.79	0	1.72	1.77	1.89	1.65	2.42
(E)-2-Octenal	2548-87-0	0.08[H]	fatty, perfume-like	31.12	2.92	7.46	4.79	100	0	28.48	0	63.69	83.95	1.97	66.17	89.58
Benzeneacetaldehyde	122-78-1	4[I]	sweet, flowery, honey-like	0.32	0.05	0	0	0	2.61	0	0	0	0	0	0	0
n-Decanal	112-31-2	2[H]	orange peel-like	0.22	0.06	0.74	0.03	0.52	3.32	0.09	0.6	0.17	0.83	0.21	0.2	0.19
Hexanal	66-25-1	2.4	green, grassy	100	6.58	19.74	1.74	12.09	0	2.52	0	6.91	0	4.84	0	7.74
Neral	106-26-3	30[J]	fatty, citrus-like	0.03	0	0	0	0	0	0	0	0	0	0.01	0	0
Nonanal	124-19-6	1[H]	green, oily	2.67	2.3	9.53	0.86	6.15	100	1.12	22.25	1.82	4.54	1.41	4.89	1.96
(E)-2-Pentenal	1576-87-0	NF	fruity	-	-	-	-	-	-	-	-	-	-	-	-	-
(E)-2-Undecenal	53448-07-0	0.78	soapy, metallic	0	0.05	6.54	0.05	3.26	0	0.49	0	0.77	2.97	1.18	1.7	1.33
Octanal	124-13-0	0.34	citrus-like, green	0	0	0	0	0	0	2.4	0	4.53	0	5.07	0	7.82
2,6-(E,Z)-Nonadienal	557-48-2	0.0045	cucumber-like	0	100	0	100	45.86	0	0	0	33.01	100	60.69	0	57.94
(E,E)-2,4-Decadienal	25152-84-5	0.027	fatty, deep-fried	0	0	0	1.66	21.71	0	0	36.26	0	0	0	38.11	0
2,4-Decadienal	2363-88-4	NF	Coriander, Deep Fried, Fat, Oil, Oxidized	-	-	-	-	-	-	-	-	-	-	-	-	-
**Acids**																
Octanoic acid	124-07-2	6	carrot-like, musty	0	0	0	0	0	2.03	0.02	0.75	0.06	0.55	0.04	0.22	0
Nonanoic acid	112-05-0	NF	moldy, pungent	-	-	-	-	-	-	-	-	-	-	-	-	-
**Esters**																
Methyl nonanoate	1731-84-6	NF	Coconut, Floral, Fruit	-	-	-	-	-	-	-	-	-	-	-	-	-
Ethyl 3-	2021-28-5	2.1	cinnamon-like, fruity	0	0	0.18	0	0.12	0	0.01	0.24	0	0	0	0	0

phenylpropionate																
Ethyl caprylate	106-32-1	8.7	fruity, green	0	0	0.19	0	0	0	0	0	0	0	0	0	0
γ-Undecalactone	104-67-6	2.1	Peach-like	0.01	0	0	0	0	0	0	0	0	0	0	0.01	0
**Sulfides**																
Allyl Isothiocyanate	57-06-7	NF	Garlic, Pungent, Sulfur	-	-	-	-	-	-	-	-	-	-	-	-	-
2-isothiocyanatobutane	4426-79-3	100[K]	Green	0	0	0	0	0.01	0.17	0	0.03	0	0	0	0	0
1-Isothiocyanatobutane	592-82-5	NF	Green, Pungent, Sulfur	-	-	-	-	-	-	-	-	-	-	-	-	-
3-Methylthiopropyl isothiocyanate	505-79-3	NF	sulfuric, garlic-like, mushroom-like	-	-	-	-	-	-	-	-	-	-	-	-	-
Phenethyl isothiocyanate	2257-09-2	NF	boiled turnip-like	-	-	-	-	-	-	-	-	-	-	-	-	-
3-Butenyl isothiocyanate	3386-97-8	17[L]	pungent, garlic-like	0	0	0	0	0	13.2	0	0.54	0	0	0	0	0
Benzyl Isothiocyanate	622-78-6	NF	herb-like, sweaty, pungent	-	-	-	-	-	-	-	-	-	-	-	-	-
Erucin	4430-36-8	NF	mushroom-like, broth-like	-	-	-	-	-	-	-	-	-	-	-	-	-

**Others**																
5-Methylthiopentanonitrile	59121-25-4	31000	broccoli-like, cabbage-like	0	0	0	0	0	0	0	0	0	0	0	0	0
Benzenepropanenitrile	645-59-0	NF	sour, fatty	-	-	-	-	-	-	-	-	-	-	-	-	-
Benzeneacetonitrile	140-29-4	NF	mushroom-like	-	-	-	-	-	-	-	-	-	-	-	-	-

[a] NF indicates that the reference retention index corresponding to the substance was not found. The odor threshold is the threshold in the water medium, and obtained from threshold databases and the literature. The odor threshold and odor description are based on Leibniz Institute for Food Systems Biology at the Technical University of Munich (Leibniz-LSB@TUM) (https://www.leibniz-lsb.de/en/) and literature from Flavor and Extract Manufacturers Association of the United States (FEMA) (https://www.femaflavor.org/).

[A] Xue, B., Li, G., Xun, X. J., Huang, Q., & Wang, S. K. (2024). Multi-omics revealed the formation mechanism of characteristic volatiles in Tibetan yak cheese induced by different altitudes. *Food* Chemistry-*X*, *21*, Article 101120. https://doi:10.1016/j.fochx.2024.101120

[B] Xia, D., Tan, X., Wang, L., Li, Z. J., Hou, A. X., Zhu, Y., Lai, L., & Wang, Y. L. (2023). GC-MS coupled with Rate-All-That-Apply (RATA) to analyse the volatile flavor substances of yellow wine during fermentation. *Foods*, *12*(10), Article 1992. https://doi:10.3390/foods12101992

[C] Shi, L. F., Wang, Q., Xie, Z. Z., Wu, C. X., Peng, T. T., Nian, X. R., Li, L., Li, H., & Chen, T. (2024). The changes of fungal community and flavor substances in Yunnan-style sausages: A comparative analysis of different drying methods. *Food Chemistry*, *460*(3), Article 140750. https://doi:10.1016/j.foodchem.2024.140750

[D] Pino, J. A. (2012). Analysis of odour-active compounds of black mangrove (*Avicennia germinans* L.) honey by solid-phase microextraction combined with gas chromatography-mass spectrometry and gas chromatography-olfactometry. *International Journal of Food Science and Technology*, *47*(8), 1688-1694. https://doi:10.1111/j.1365-2621.2012.03021.x

[E] Zhao, Y., Zhang, R. G., He, W. Y., Geng, J. Z., Wang, P., Tian, H. L., & Zhan, P. (2025). Characterization of aroma release and perception during ginger-infused stewed beef oral processing. *Food Chemistry*, *475*, Article 143155. https://doi:10.1016/j.foodchem.2025.143155

[F] Ou, Z. J., Chang, S. Y., Li, S. C., Tan, Y. L., Zhou, Y. N., & Pang, X. L. (2024). Characterization of the key aroma compounds in different varieties of hops by application of the Sensomics approach. *Food Chemistry*, *460*(2), Article 140448. https://doi:10.1016/j.foodchem.2024.140448

[G] Wang, Y. B., Wu, D., Wu, Y. Q., Tong, X. Q., Qin, Y. C., & Wang, L. L. (2023). Difference in volatile aroma components of *Stropharia rugosoannulata* under two cultivated environments investigated by SPME-GC-MS. *Foods*, *12*(14), Article 2656. https://doi:10.3390/foods12142656

[H] Xue, B., Li, G., Xun, X. J., Huang, Q., & Wang, S. K. (2024). Multi-omics revealed the formation mechanism of characteristic volatiles in Tibetan yak cheese induced by different altitudes. *Food Chemistry-X*, *21*, Article 101120. https://doi:10.1016/j.fochx.2024.101120

[I] Wang, S. Y., Su, Q. Y., Zhu, Y. X., Liu, J. N., Zhang, X. K., Zhang, Y., & Zhu, B. Q. (2024). Sensory-guided establishment of sensory lexicon and investigation of key flavor components for goji berry pulp. *Plants-Basel*, *13*(2), Article 173. https://doi:10.3390/plants13020173

[J] Pu, D. D., Shan, Y. M., Duan, W., Huang, Y., Liang, L., Yan, Y., Zhang, Y. Y., Sun, B. G., & Hu, G. H. (2021). Characterization of the key aroma compounds in the

fruit of *Litsea pungens* Hemsl. (LPH) by GC-MS/O, OAV, and Sensory Techniques. *Journal of Food Quality*, *2021*, Article 6668606. https://doi:10.1155/2021/6668606
[K] Zhou, Q., Jia, X., Yao, Y. Z., Wang, B., Wei, C. Q., Zhang, M., & Huang, F. H. (2019). Characterization of the aroma-active compounds in commercial fragrant rapeseed oils via monolithic material sorptive extraction. *Journal of Agricultural and Food Chemistry, 67*(41), 11454-11463. https://doi:10.1021/acs.jafc.9b05691
[L] Wei, S. H., Xiao, X. M., Wei, L. J., Li, L. S., Li, G. C., Liu, F. H., Xie, J. M., Yu, J. H., & Zhong, Y. (2021). Development and comprehensive HS-SPME/GC-MS analysis optimization, comparison, and evaluation of different cabbage cultivars (*Brassica oleracea* L. var. *capitata* L.) volatile components. Food Chemistry, 340, Article 128166. https://doi:10.1016/j.foodchem.2020.128166
